# Development and validation of a cross-sectional risk screening nomogram for carotid plaque based on routine health examination data and the triglyceride-glucose-waist-to-hip ratio

**DOI:** 10.3389/fendo.2026.1881365

**Published:** 2026-06-17

**Authors:** Yilian Zhang, Xu Zhang, Hongbin Song, Qiyu Hu, Jingzhu Nan

**Affiliations:** 1 Department of Clinical Laboratory, The First Medical Center of Chinese PLA, Beijing, China; 2 Department of Clinical Laboratory, Beijing Anzhen Hospital, Capital Medical University, Beijing, China; 3 Department of Clinical Laboratory, The Third Medical Center of Chinese PLA, Beijing, China

**Keywords:** carotid plaque, cross sectional risk stratification, health checkup data, logistic regression, triglyceride glucose waist to hip ratio

## Abstract

**Introduction:**

Primary prevention of atherosclerotic cardiovascular disease (ASCVD) requires the identification of high-risk individuals in the general population who may currently harbor carotid plaque (CP). Existing tools fail to adequately capture the combined impact of insulin resistance (IR) and central obesity. This study aimed to develop a cross-sectional risk stratification model for identifying prevalent carotid plaque using routine health examination data.

**Methods:**

A total of 4,992 participants from a health examination cohort constituted the development set and were randomly divided into a training set and an internal validation set (7:3 ratio). An additional temporal validation set comprising 3,812 participants was included. All variables and the outcome (presence or absence of carotid plaque) were synchronously collected within a single health examination cycle. The triglyceride-glucose-waist-to-hip ratio (TyG-WHR) was calculated. Recursive feature elimination (RFE) was employed for variable selection, and a logistic regression model was constructed in the form of a nomogram. Model performance in discriminating prevalent carotid plaque was evaluated in the internal validation set and the temporal validation set.

**Results:**

TyG-WHR was independently associated with prevalent CP, and its discriminatory performance (area under the curve [AUC]=0.689) was superior to that of the conventional TyG index (AUC=0.642). Furthermore, its incremental value over a base model composed of traditional clinical variables was significant in the temporal validation set (net reclassification improvement [NRI]=0.472, P < 0.001). The logistic regression nomogram constructed using eight routine health examination indices achieved an area under the receiver operating characteristic curve (AUROC) of 0.85 in the temporal validation set, with good calibration. In the absence of external validation, these results are intended for research purposes only and should not be directly applied in clinical practice.

**Discussion:**

This cross-sectional nomogram, developed from routine health examination data, provides a convenient auxiliary screening tool for identifying individuals in the general population who may currently harbor carotid plaque, thereby supporting risk stratification in large-scale health check-ups. It must be emphasized that this model is designed to identify individuals who may currently have subclinical carotid plaque, rather than to predict future incident events.

## 1 Introduction

Atherosclerotic cardiovascular disease (ASCVD) is the leading cause of death and disability worldwide (1–3). The carotid artery, serving as a window to the systemic arterial system (4), enables the detection of carotid plaque (CP) to provide an early and accessible marker for assessing atherosclerotic burden (5–8). Accurately identifying individuals with existing CP during the subclinical stage carries substantial public health significance for the primary prevention of ASCVD (9).

Traditional risk assessment relies on factors such as age, hypertension, diabetes mellitus, and dyslipidemia (10–12); however, these factors cannot fully account for individual risk heterogeneity-many individuals classified as intermediate-risk experience unexpected events, while some with high-risk scores remain stable over the long term. This suggests that intertwined pathogenic pathways involving metabolic dysregulation, insulin resistance (IR), and abnormal fat distribution may be overlooked (13–15).

The triglyceride-glucose (TyG) index has attracted attention owing to its strong correlation with IR (16–18); however, it fails to incorporate central obesity, which constitutes an independent risk dimension. Central obesity directly accelerates atherosclerosis through the secretion of pro-inflammatory adipokines and abnormalities in lipid metabolism (19–21). The TyG-WHR index, formed by combining the TyG index with the waist-to-hip ratio (WHR), theoretically captures two critical pathways simultaneously: IR and adverse fat distribution. Nevertheless, the performance of TyG-WHR in the cross-sectional screening for CP in the general population, as well as its incremental value over the TyG index, has yet to be adequately validated.

With the increasing application of data-driven approaches in clinical medicine (22–25), it has become feasible to construct screening tools with satisfactory discrimination, calibration, and generalizability using routine examination indices (26, 27). The present study aimed to systematically evaluate the association between TyG-WHR and prevalent carotid plaque, and to construct a cross-sectional risk screening nomogram based on routine clinical indices, thereby enabling individualized risk stratification in a simple and intuitive manner to guide clinical decision-making regarding carotid ultrasound examination.

## 2 Materials and methods

### 2.1 Study design and population

**Figure 1:**
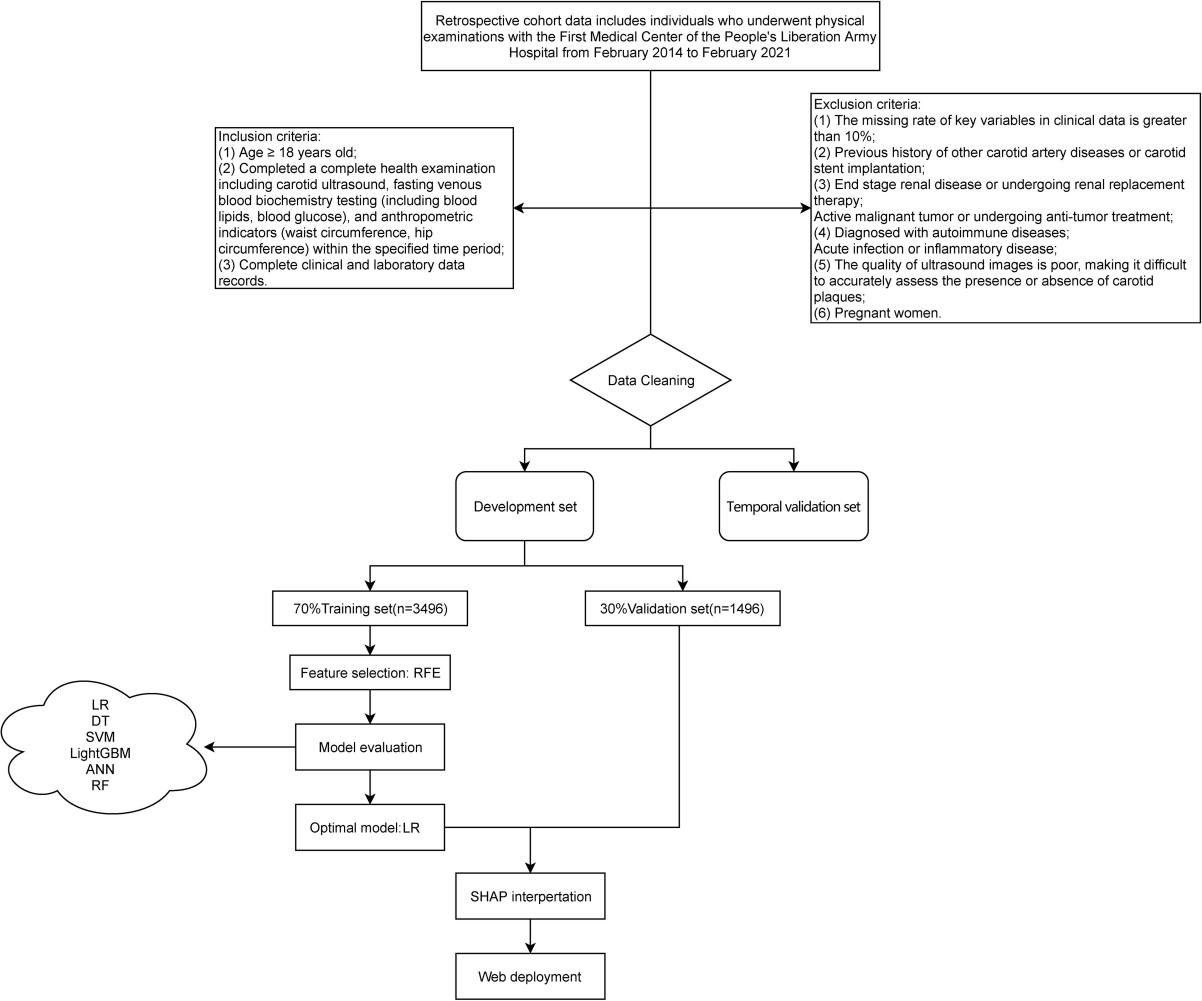
The flowchart of this study.

This was a retrospective observational study designed to evaluate factors associated with prevalent carotid plaque in the general population using the TyG-WHR index, and to construct a cross-sectional risk screening model. Data were derived from the indices of individuals who underwent health examinations and received a first-time diagnosis of carotid plaque at the First Medical Center of the Chinese People’s Liberation Army (PLA) General Hospital. It should be noted that this study employed a cross-sectional risk stratification design. All predictor variables were derived from baseline health examination data, and the outcome (presence or absence of carotid plaque) was synchronously assessed via carotid ultrasound within the same health examination cycle. Therefore, it must be explicitly emphasized that this model is designed for the screening and risk stratification of prevalent carotid plaque in the general health examination population, rather than for predicting whether an individual will newly develop carotid plaque in the future. This cross-sectional nature must be fully appreciated when applying the model. The development set included 4,992 individuals who completed carotid ultrasound examination between February 2014 and March 2017, comprising 1,549 cases in the CP group and 3,443 cases in the non-carotid plaque (Non-CP) group. The development set was randomly divided into a training set (n=3,496) and an internal validation set (n=1,496) at a 7:3 ratio. An additional 3,812 individuals who underwent examination between March 2020 and February 2021 were enrolled as an independent temporal validation set to assess the model’s generalizability. The inclusion and exclusion criteria are presented in **Figure 1**. This study was approved by the Medical Ethics Committee of the Chinese PLA General Hospital (ethics approval number: S2025-987-01).

### 2.2 Variable definitions and data preprocessing

The TyG-WHR index was calculated using the following formula: TyG-WHR=Ln [fasting triglyceride (TG) (mg/dL) × fasting glucose (Glu) (mg/dL)/2] × waist circumference (WC) (cm)/hip circumference (HC) (cm).

All carotid ultrasound examinations were performed using the same model of color Doppler ultrasound system. The examinations were conducted by three attending physicians with more than five years of experience in vascular ultrasound. Prior to the commencement of the study, standardized training was provided to ensure consistency in scanning protocols, measurement techniques, and diagnostic criteria. Throughout the examination and image interpretation process, the sonographers remained blinded to the participants’ clinical data (including medical history and laboratory test results). Carotid plaque was defined in accordance with the Mannheim Carotid Intima-Media Thickness and Plaque Consensus: a focal structure encroaching into the arterial lumen by at least 0.5 mm or exceeding 50% of the surrounding intima-media thickness (IMT) value, or a focal region with an IMT ≥ 1.5 mm measured from the intima-lumen interface to the media-adventitia interface. In addition, the number of plaques (single/multiple) and their locations (left/right side; common carotid artery/bifurcation/internal carotid artery) were systematically recorded. Although the primary outcome of this study was the presence or absence of carotid plaque, these detailed plaque characteristics have been archived and will be utilized in subsequent secondary analyses (e.g., stratification of plaque vulnerability or progression risk), thereby facilitating further exploration of the association between sonographic plaque features and metabolic biomarkers. Variables with a missing rate exceeding 10% were excluded. For variables with a missing rate ≤ 10%, multiple imputation was performed using the random forest algorithm implemented in the mice package in R. The proportions of missing data are presented in **Supplementary Table 1**. Random forest multiple imputation (R mice package) was conducted exclusively within the training set at each modeling stage. For cross-validation, imputation was performed independently within the training set of each fold; the imputation model parameters were learned solely from that training set and subsequently applied to the corresponding validation fold. For the independent temporal validation set, the imputation model parameters were obtained from the complete training set and applied in a single step.

### 2.3 Statistical Analysis

Data analysis was performed using R 4.5.2 (for statistical analysis and imputation) and Python 3.12 (for machine learning [ML] modeling). Continuous variables were expressed as median (interquartile range [IQR]), and categorical variables were expressed as frequency (percentage). Between-group comparisons were conducted using the Mann-Whitney U test or the chi-squared test. Multivariable logistic regression was used to analyze the association between TyG-WHR and prevalent carotid plaque. Odds ratios (ORs) and 95% confidence intervals (CIs) were calculated for TyG-WHR both as a continuous variable (per standard deviation [SD] increase) and by quartile groups (Q1-Q4), and the P-for-trend was reported. Subgroup analyses assessed heterogeneity across different populations (e.g., stratified by sex, age, and comorbidities). Restricted cubic spline (RCS) analysis explored the dose-response relationship between TyG-WHR and prevalent carotid plaque, and the P-for-nonlinearity was reported. Receiver operating characteristic (ROC) curve analysis was employed to assess discriminatory ability, with the area under the curve (AUC), optimal cutoff value, sensitivity, and specificity reported. The significance level was set at P < 0.05.

### 2.4 Sample size calculation

The number of carotid plaque events in the training set was 1,085, yielding an events-per-variable (EPV) ratio of 1,085/56≈19.4, which far exceeded the conventional rule-of-thumb criterion of EPV ≥ 10, indicating that the sample size of this study was adequate to support the subsequent variable screening and model construction. Moreover, the eight-variable final model of this study (EPV≈136) substantially exceeded this criterion.

### 2.5 Construction of the risk screening model and nomogram development

To ensure fairness in model evaluation and to prevent data leakage, all data processing and feature selection steps related to model construction in this study were strictly conducted within the training set. Collinearity analysis was performed exclusively on the imputed training set, and highly correlated variables were identified and removed based on the correlation coefficient matrix to mitigate the risk of multicollinearity. Based on the relatively independent variables retained after this screening, recursive feature elimination (RFE) was again conducted exclusively within the training set. The RFE process was combined with a random forest classifier, and the performance of different feature subsets was evaluated via five-fold cross-validation to determine the optimal core feature subset. This step aimed to reduce data dimensionality and model complexity, thereby enhancing the model’s generalizability and interpretability. Ultimately, the feature subset selected by RFE was designated as the final variable set for model construction. Based on these selected variables, seven ML models were trained on the training set, and their hyperparameters were optimized via grid search combined with five-fold cross-validation. Throughout the entire process, the data from the internal validation set and the temporal validation set were used exclusively for the final evaluation of model performance and did not participate in any feature selection or parameter tuning steps. This rigorous workflow ensured the authenticity of the validation results and the credibility of the model’s generalizability. Logistic regression was ultimately chosen as the final model and was visualized in the form of a nomogram to facilitate direct clinical use. The Hosmer-Lemeshow goodness-of-fit test and the Brier Score were employed to assess the calibration of the nomogram. The selection of cutoff values took into consideration the intended role of this nomogram as a preliminary screening tool in health examinations. In addition to reporting the cutoff value that maximized Youden’s index, a high-sensitivity screening cutoff (predicted probability=0.25) was determined on the training set with the objective of achieving a sensitivity ≥ 0.80, in order to minimize missed diagnoses. Furthermore, subgroup analyses stratified by sex, age, and comorbidities were conducted to delineate the applicable population boundaries of the model.

## 3 Results

### 3.1 Baseline characteristics of the study population

The development set included a total of 4,992 individuals who completed carotid ultrasound assessment. Among them, 1,549 cases (31.03%) had CP (CP group) and 3,443 cases (68.97%) had no CP (Non-CP group). The median age of the total population was 50.0 years (IQR: 45.0-56.0), and 68.65% were male.

Compared with the Non-CP group, the CP group was older (55.0 years vs. 48.0 years, P < 0.001) and had significantly higher prevalence rates of hypertension (40.93% vs. 28.26%, P < 0.001) and diabetes mellitus (31.83% vs. 19.05%, P < 0.001).

Regarding metabolic and body fat distribution indices, the CP group exhibited more adverse levels across multiple measures, including higher systolic blood pressure (SBP), fasting glucose, glycated hemoglobin (HbA1c), triglycerides, and several composite indices related to IR (all P < 0.001). With respect to inflammation and coagulation, the CP group had significantly higher levels of the neutrophil-to-lymphocyte ratio (NLR), monocyte-to-lymphocyte ratio (MLR), and fibrinogen (FIB) (all P < 0.05). Additionally, indices reflecting renal function and uric acid metabolism also demonstrated significant differences (all P < 0.001). Detailed baseline characteristics are presented in **Table 1**.

**Table 1:** Baseline characteristics of the overall study population.

Variables	Total(n=4992)	Non-CP(n=3443)	CP(n=1549)	P
Hypertension	1607 (32.19)	973 (28.26)	634 (40.93)	<0.001
DM	1149 (23.02)	656 (19.05)	493 (31.83)	<0.001
Sex				0.007
Male	3427 (68.65)	2405 (69.85)	1022 (65.98)	
Female	1565 (31.35)	1038 (30.15)	527 (34.02)	
Age	50.00 [45.00, 56.00]	48.00 [42.00, 53.00]	55.00 [51.00, 61.00]	<0.001
BMI	25.10 [22.90, 27.20]	24.70 [22.50, 26.90]	25.70 [23.80, 27.80]	<0.001
MM (Kg)	48.80 [40.40, 54.30]	48.30 [39.70, 53.90]	49.90 [43.30, 55.00]	<0.001
WC(cm)	87.00 [81.00, 94.00]	86.00 [79.00, 93.00]	90.00 [84.00, 96.00]	<0.001
WHR	0.92 [0.84, 0.96]	0.91 [0.83, 0.95]	0.94 [0.89, 0.98]	<0.001
Protein(g/dL)	10.70 [8.70, 11.90]	10.60 [8.60, 11.80]	10.90 [9.30, 12.00]	<0.001
SBP(mmHg)	124.00 [114.00, 134.00]	122.00 [112.00, 131.00]	129.00 [119.00, 139.00]	<0.001
DBP(mmHg)	84.00 [76.00, 91.00]	83.00 [74.00, 90.00]	86.00 [79.00, 92.00]	<0.001
NLR	1.79 [1.41, 2.26]	1.77 [1.41, 2.24]	1.83 [1.43, 2.31]	0.036
MLR	0.17 [0.14, 0.21]	0.17 [0.14, 0.21]	0.18 [0.14, 0.22]	<0.001
PLR	123.79 [99.99, 150.95]	125.65 [102.11, 152.24]	119.05 [95.35, 148.04]	<0.001
WBC(×10^9/L)	5.70 [4.90, 6.73]	5.66 [4.85, 6.64]	5.83 [5.00, 6.91]	<0.001
NEUT(×10^9/L)	3.30 [2.70, 4.07]	3.27 [2.68, 4.00]	3.40 [2.73, 4.24]	<0.001
LYM(×10^9/L)	1.84 [1.53, 2.22]	1.84 [1.52, 2.20]	1.86 [1.54, 2.25]	0.107
MONO(×10^9/L)	0.32 [0.26, 0.39]	0.31 [0.25, 0.38]	0.33 [0.27, 0.40]	<0.001
PLT(×10^9/L)	228.00 [195.00, 263.00]	230.00 [198.00, 265.00]	221.00 [190.00, 258.00]	<0.001
RBC(×10^12/L)	4.82 [4.49, 5.12]	4.82 [4.47, 5.12]	4.83 [4.54, 5.12]	0.09
Hb(g/L)	148.00 [137.00, 157.00]	148.00 [135.00, 156.50]	149.00 [140.00, 157.00]	<0.001
MCV(fL)	89.30 [87.00, 91.60]	89.10 [86.90, 91.50]	89.60 [87.40, 92.00]	<0.001
MCHC(g/L)	342.00 [336.00, 348.00]	342.00 [335.00, 348.00]	342.00 [336.00, 349.00]	0.002
MCH(pg)	30.60 [29.70, 31.50]	30.50 [29.60, 31.40]	30.70 [29.80, 31.70]	<0.001
MPV(fL)	10.20 [9.70, 10.80]	10.20 [9.70, 10.90]	10.20 [9.60, 10.80]	0.098
RDW-CV(%)	12.30 [11.90, 12.70]	12.30 [11.90, 12.70]	12.30 [11.90, 12.70]	0.43
Hct	0.43 [0.40, 0.46]	0.43 [0.40, 0.46]	0.43 [0.41, 0.46]	<0.001
INR	0.95 [0.91, 0.99]	0.95 [0.91, 0.99]	0.94 [0.91, 0.99]	0.187
PT(Sec)	12.70 [12.30, 13.10]	12.70 [12.30, 13.10]	12.60 [12.20, 13.10]	0.207
PA	109.00 [102.00, 118.00]	109.00 [102.00, 118.00]	111.00 [102.00, 120.00]	0.174
APTT(Sec)	34.60 [32.60, 37.00]	34.70 [32.60, 37.00]	34.50 [32.50, 36.70]	0.017
FIB(g/L)	3.04 [2.73, 3.42]	3.01 [2.69, 3.35]	3.14 [2.80, 3.59]	<0.001
PHR	181.57 [143.54, 227.43]	181.69 [142.91, 227.66]	181.37 [145.00, 227.00]	0.699
MHR	0.26 [0.19, 0.34]	0.25 [0.18, 0.33]	0.28 [0.20, 0.36]	<0.001
TG(mmol/L)	1.42 [1.00, 2.11]	1.36 [0.96, 2.07]	1.54 [1.11, 2.19]	<0.001
CHO(mmol/L)	4.65 [4.09, 5.24]	4.62 [4.10, 5.19]	4.73 [4.07, 5.37]	0.014
HDL-C(mmol/L)	1.25 [1.04, 1.50]	1.26 [1.05, 1.52]	1.22 [1.03, 1.44]	<0.001
LDL-C(mmol/L)	3.00 [2.50, 3.54]	2.97 [2.50, 3.48]	3.08 [2.47, 3.66]	0.001
nonHDL-C(mmol/L)	3.35 [2.81, 3.93]	3.32 [2.80, 3.89]	3.46 [2.83, 4.10]	<0.001
RLP-C(mmol/L)	0.23 [0.10, 0.46]	0.22 [0.09, 0.44]	0.26 [0.13, 0.48]	<0.001
HbA1c	5.80 [5.60, 6.10]	5.70 [5.50, 6.00]	6.00 [5.70, 6.30]	<0.001
Glu(mmol/L)	5.47 [5.12, 5.94]	5.38 [5.06, 5.80]	5.70 [5.27, 6.34]	<0.001
ALT(U/L)	19.10 [13.80, 28.00]	18.60 [13.40, 27.70]	20.00 [14.90, 28.60]	<0.001
AST(U/L)	18.30 [15.50, 22.10]	18.00 [15.30, 21.80]	19.00 [16.10, 22.80]	<0.001
ALP(U/L)	62.40 [52.70, 74.80]	61.40 [52.00, 73.30]	65.10 [55.00, 77.80]	<0.001
GGT(U/L)	26.10 [16.60, 44.12]	25.10 [15.60, 43.40]	27.90 [19.00, 46.30]	<0.001
TP(g/L)	73.00 [70.40, 75.60]	73.10 [70.50, 75.70]	72.80 [70.20, 75.30]	0.034
ALB(g/L)	46.40 [44.80, 48.10]	46.50 [44.80, 48.10]	46.30 [44.70, 47.90]	0.021
TB(umol/L)	11.50 [8.70, 15.20]	11.30 [8.50, 15.00]	12.00 [9.10, 15.50]	<0.001
DB(umol/L)	3.90 [3.10, 4.90]	3.90 [3.00, 4.90]	4.00 [3.20, 5.10]	<0.001
BUN(mmol/L)	4.95 [4.23, 5.79]	4.89 [4.16, 5.71]	5.11 [4.38, 5.95]	<0.001
Cr(mg/dL)	0.81 [0.69, 0.92]	0.80 [0.68, 0.92]	0.83 [0.72, 0.93]	<0.001
CREA(umol/L)	71.30 [60.80, 81.30]	70.30 [59.80, 81.10]	73.10 [63.40, 82.10]	<0.001
eGFR(mL/min/1.73m^2)	101.37 [93.11, 107.88]	103.00 [94.66, 109.61]	98.01 [90.04, 103.91]	<0.001
UA(umol/L)	342.30 [281.50, 404.42]	336.70 [275.75, 400.90]	354.20 [297.20, 411.10]	<0.001
Ca(mmol/L)	2.34 [2.29, 2.39]	2.34 [2.28, 2.39]	2.34 [2.29, 2.39]	0.112
PHOS(mmol/L)	1.16 [1.07, 1.26]	1.16 [1.07, 1.26]	1.17 [1.07, 1.26]	0.904
K(mmol/L)	4.32 [4.14, 4.52]	4.31 [4.13, 4.51]	4.35 [4.15, 4.53]	0.007
Na(mmol/L)	142.00 [141.00, 144.00]	142.00 [140.00, 144.00]	143.00 [141.00, 144.00]	<0.001
Cl(mmol/L)	101.50 [99.90, 103.10]	101.60 [99.80, 103.20]	101.40 [99.90, 103.10]	0.224
UACR(mg/g)	5.60 [4.00, 9.20]	5.30 [3.90, 8.60]	6.40 [4.30, 11.60]	<0.001
TgAb(IU/mL)	12.00 [10.10, 15.10]	12.10 [10.20, 15.20]	11.80 [10.00, 14.90]	0.027
TPOAb(IU/mL)	13.40 [9.90, 17.80]	13.40 [9.90, 17.80]	13.60 [9.90, 17.80]	0.501
T3(nmol/L)	1.77 [1.58, 1.96]	1.76 [1.58, 1.96]	1.78 [1.60, 1.99]	0.018
TSH(mIU/l)	2.08 [1.46, 3.00]	2.10 [1.48, 3.00]	2.00 [1.40, 2.99]	0.01
FT3(pmol/L)	5.08 [4.67, 5.53]	5.08 [4.66, 5.54]	5.08 [4.70, 5.50]	0.898
FT4(pmol/L)	16.82 [15.43, 18.43]	16.89 [15.42, 18.44]	16.69 [15.47, 18.32]	0.124
T4(nmol/L)	97.16 [86.39, 108.30]	96.93 [86.24, 107.90]	97.58 [86.56, 109.30]	0.068
HCY(umol/L)	10.50 [8.70, 12.90]	10.20 [8.40, 12.60]	11.20 [9.30, 13.40]	<0.001
hs-CRP(mg/L)	0.08 [0.05, 0.15]	0.08 [0.04, 0.14]	0.09 [0.05, 0.18]	<0.001
sd-LDL-C(mmol/L)	9.69 [9.45, 9.92]	9.68 [9.45, 9.91]	9.70 [9.44, 9.97]	0.105
TyG	8.75 [8.36, 9.20]	8.70 [8.29, 9.16]	8.89 [8.51, 9.30]	<0.001
ASBI	0.06 [0.06, 0.07]	0.06 [0.06, 0.07]	0.06 [0.06, 0.06]	0.775
CHG	5.23 [4.98, 5.46]	5.19 [4.95, 5.42]	5.30 [5.07, 5.53]	<0.001
NHHR	2.70 [2.02, 3.46]	2.65 [1.97, 3.38]	2.81 [2.13, 3.59]	<0.001
RCII	0.08 [0.02, 0.22]	0.07 [0.02, 0.20]	0.10 [0.03, 0.26]	<0.001
CALLY	105.35 [56.21, 182.65]	111.21 [59.56, 192.97]	92.53 [49.80, 159.17]	<0.001
CLR	0.04 [0.03, 0.08]	0.04 [0.02, 0.08]	0.05 [0.03, 0.09]	<0.001
dNLR	0.88 [0.85, 0.90]	0.88 [0.85, 0.90]	0.87 [0.84, 0.89]	0.002
NMLR	1.96 [1.57, 2.47]	1.94 [1.56, 2.44]	2.01 [1.58, 2.51]	0.021
SIRI	0.56 [0.41, 0.80]	0.55 [0.40, 0.77]	0.59 [0.42, 0.85]	<0.001
SII	406.27 [305.24, 535.36]	406.09 [308.57, 534.21]	406.55 [298.34, 538.02]	0.31
SHR	0.83 [0.78, 0.89]	0.83 [0.78, 0.89]	0.83 [0.78, 0.90]	0.238
TyG-WHR	8.06 [7.26, 8.73]	7.92 [7.05, 8.60]	8.35 [7.75, 8.97]	<0.001

CP, Carotid Plaque; Non-CP, Non-Carotid Plaque; BMI, Body Mass Index; DM, Diabetes Mellitus; MM, Muscle Mass; BMR, Basal Metabolic Rate; WC, Waist Circumference; WHR, Waist-to-Hip Ratio; SBP, Systolic Blood Pressure; DBP, Diastolic Blood Pressure; NLR, Neutrophil-to-Lymphocyte Ratio; MLR, Monocyte-to-Lymphocyte Ratio; PLR, Platelet-to-Lymphocyte Ratio; WBC, White Blood Cell Count; NEUT, Neutrophil Count; LYM, Lymphocyte Count; MONO, Monocyte Count; PLT, Platelet Count; RBC, Red Blood Cell Count; Hb, Hemoglobin; MCV, Mean Corpuscular Volume; MCHC, Mean Corpuscular Hemoglobin Concentration; MCH, Mean Corpuscular Hemoglobin; MPV, Mean Platelet Volume; RDW, Red Cell Distribution Width; Hct, Hematocrit; INR, International Normalized Ratio; PT, Prothrombin Time; PA, Prothrombin Activity; APTT, Activated Partial Thromboplastin Time; FIB, Fibrinogen; PHR, Platelet-to-Hemoglobin Ratio; MHR, Monocyte-to-High-density lipoprotein Cholesterol Ratio; TG, Triglyceride; CHO, Total Cholesterol; HDL-C, High-density Lipoprotein Cholesterol; LDL-C, Low-density Lipoprotein Cholesterol; nonHDL-C, Non-High-Density Lipoprotein Cholesterol; RLP-C, Remnant Lipoprotein Cholesterol; HbA1c, Glycated Hemoglobin; Glu, Glucose; ALT, Alanine Aminotransferase; AST, Aspartate Aminotransferase; ALP, Alkaline Phosphatase; GGT, Gamma-Glutamyl Transferase; TP, Total Protein; ALB, Albumin; TB, Total Bilirubin; DB, Direct Bilirubin; BUN, Blood Urea Nitrogen; Cr, Creatinine; CREA, Creatinine; eGFR, Estimated Glomerular Filtration Rate; UA, Uric Acid; Ca, Calcium; PHOS, Phosphorus; K, Potassium; Na, Sodium; Cl, Chloride; UACR, Urine Albumin-to-Creatinine Ratio; TgAb, Thyroglobulin Antibody; TPOAb, Thyroid Peroxidase Antibody; T3, Triiodothyronine; TSH, Thyroid Stimulating Hormone; FT3, Free Triiodothyronine; FT4, Free Thyroxine; T4, Thyroxine; HCY, Homocysteine; hs-CRP, High-sensitivity C-reactive Protein; sd-LDL-C, Small Dense Low-Density Lipoprotein Cholesterol; TyG, Triglyceride-Glucose Index; ASBI, Atherogenic Index of Plasma; CHG, Cholesterol, high-density lipoprotein, and glucose index; NHHR, Non-HDL-C to HDL-C Ratio; RCII, Residual Cholesterol Ischemic Index; CALLY, C-Reactive Protein-Albumin-Lymphocyte Ratio; CLR, C-Reactive Protein to Lymphocyte Ratio; dNLR, Derived Neutrophil-to-Lymphocyte Ratio; NMLR, Neutrophil-Monocyte-to-Lymphocyte Ratio; SIRI, Systemic Inflammatory Response Index; SII, Systemic Immune-Inflammation Index; SHR, Stress Hyperglycemia Ratio; TyG-WHR, Triglyceride-Glucose-Waist-to-Hip Ratio;.

To ensure reliable model development and validation, the entire population (n=4,992) was randomly divided into a training set (n=3,496) and an internal validation set (n=1,496) at a 7:3 ratio. Furthermore, an independent temporal validation set (n=3,812) was included to assess generalizability. The detailed baseline characteristics of the training set and validation sets are provided in the **Supplementary Materials** (**Supplementary Tables 2**, **S3**, **S4**).

### 3.2 Association of TyG-WHR and TyG Index with carotid plaque

As shown in **Table 2**, after adjusting for demographic characteristics, body mass index (BMI), and major cardiometabolic risk factors (Model 3), individuals in the highest TyG-WHR quartile (Q4) had a 2.404-fold higher risk of CP compared with those in the lowest quartile (Q1) in the development set (95% CI: 1.854-3.116, P < 0.001). This association strength was markedly greater than that of the TyG index (Q4 vs. Q1: OR=1.390, 95% CI: 1.119-1.726, P=0.003). This trend was replicated and was even more pronounced in the independent temporal validation set: the Q4 group of TyG-WHR had a 3.814-fold higher risk than the Q1 group (95% CI: 2.626-5.542, P < 0.001), whereas the OR for the TyG index was 1.684 (95% CI: 1.293-2.194, P < 0.001). The per-SD increase analysis similarly demonstrated that the association of TyG-WHR with CP risk (development set: OR=1.394; validation set: OR=1.720) was stronger than that of the TyG index (development set: OR=1.120; validation set: OR=1.224).

**Table 2:** Association of TyG and TyG-WHR with carotid plaque in the development cohort and temporal validation cohort.

Model	model1 OR (95%CI)	P	model2 OR (95%CI)	P	model3 OR (95%CI)	P
Development set						
TyG						
Q1	reference		reference		reference	
Q2	1.813(1.510-2.177)	<0.001	1.194(0.971-1.468)	0.093	1.153(0.935-1.42)	0.183
Q3	2.153(1.796-2.580)	<0.001	1.354(1.098-1.688)	0.005	1.279(1.034-1.582)	0.023
Q4	2.332(1.948-2.792)	<0.001	1.527(1.235-1.887)	<0.001	1.390(1.119-1.726)	0.003
PerSD	1.315(1.238-1.396)	<0.001	1.160(1.078-1.249)	<0.001	1.120(1.038-1.208)	0.003
TyG-WHR						
Q1	reference		reference		reference	
Q2	2.334(1.921-2.836)	<0.001	1.564(1.250-1.957)	<0.001	1.556(1.240-1.954)	<0.001
Q3	3.100(2.559-3.755)	<0.001	2.182(1.731-2.750)	<0.001	2.188(1.724-2.777)	<0.001
Q4	3.915(3.238-4.735)	<0.001	2.423(1.885-3.115)	<0.001	2.404(1.854-3.116)	<0.001
PerSD	1.640(1.537-1.750)	<0.001	1.407(1.281-1.547)	<0.001	1.394(1.264-1.537)	<0.001
Validation set						
**TyG**						
Q1	reference		reference		reference	
Q2	1.979(1.643-2.384)	<0.001	1.203(0.954-1.518)	0.119	1.092(0.857-1.390)	0.477
Q3	2.996(2.486-3.611)	<0.001	1.761(1.392-2.228)	<0.001	1.558(1.221-1.989)	<0.001
Q4	3.672(3.039-4.437)	<0.001	2.484(1.947-3.169)	<0.001	1.684(1.293-2.194)	<0.001
PerSD	1.618(1.508-1.736)	<0.001	1.425(1.307-1.555)	<0.001	1.224(1.111-1.348)	<0.001
TyG-WHR						
Q1	reference		reference		reference	
Q2	3.369(2.771-4.096)	<0.001	2.194(1.690-2.850)	<0.001	1.873(1.425-2.461)	<0.001
Q3	4.480(3.681-5.453)	<0.001	3.632(2.645-4.986)	<0.001	3.087(2.214-4.303)	<0.001
Q4	6.760(5.527-8.268)	<0.001	5.406(3.810-7.671)	<0.001	3.814(2.626-5.542)	<0.001
PerSD	2.117(1.964-2.283)	<0.001	2.003(1.744-2.301)	<0.001	1.720(1.479-2.001)	<0.001

Model 1: Unadjusted. Model 2: Adjusted for sex, age, and body mass index (BMI). Model 3: Further adjusted for history of hypertension, history of diabetes mellitus, systolic blood pressure (SBP), and diastolic blood pressure (DBP) based on Model 2. TyG, triglyceride-glucose index; TyG-WHR, triglyceride-glucose-waist-to-hip ratio index; OR, odds ratio; CI, confidence interval; Q, quartile; SD, standard deviation.

### 3.3 Subgroup analysis of the association of TyG-WHR and TyG index with carotid plaque

**Figure 2:**
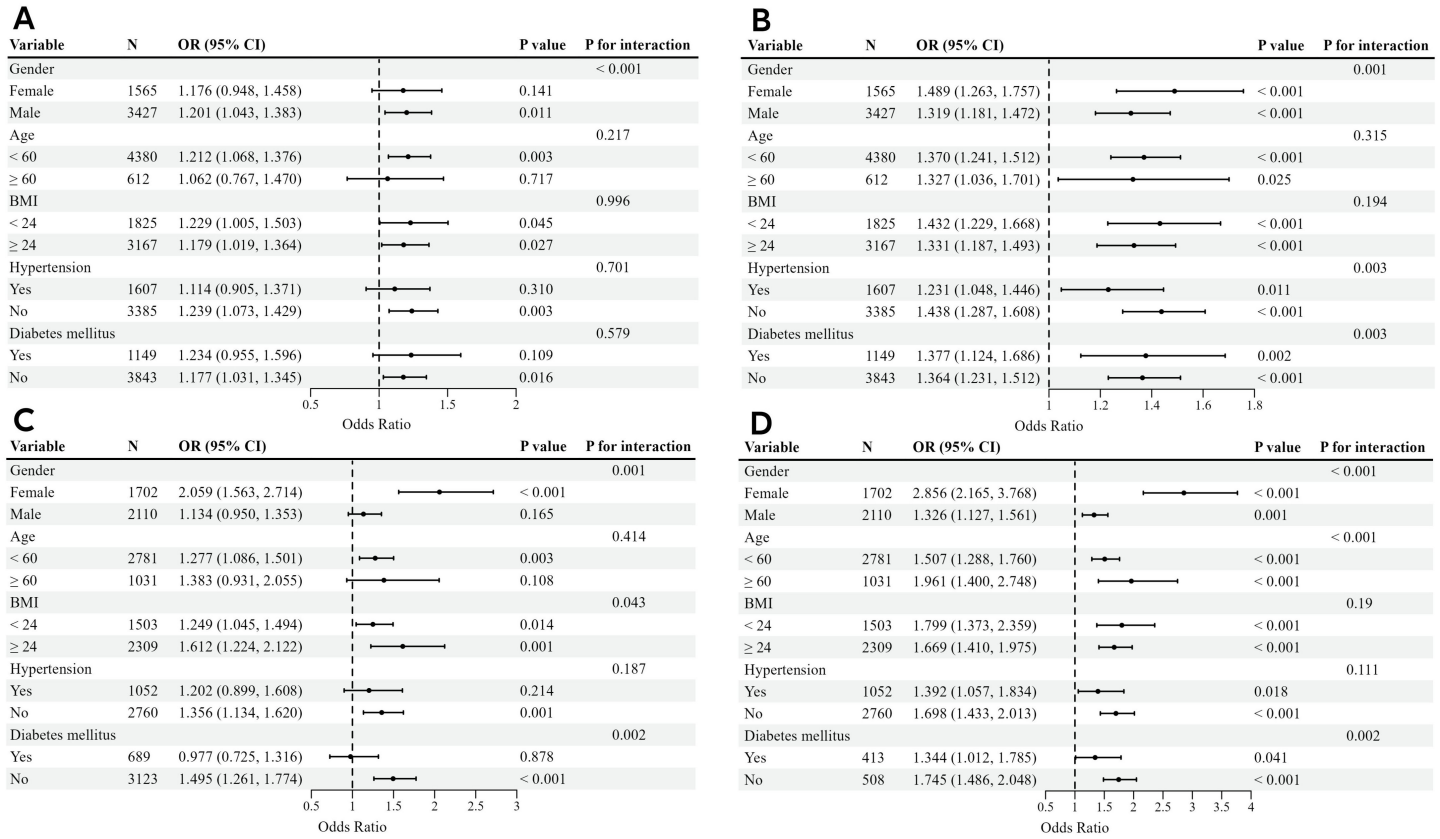
Subgroup analyses of the associations between TyG/TyG-WHR and carotid plaque. **(A)** TyG index in the development cohort. **(B)** TyG-WHR index in the development cohort. **(C)** TyG index in the temporal validation cohort. **(D)** TyG-WHR index in the temporal validation cohort. Notes: All models were adjusted for sex, age, body mass index (BMI), history of hypertension, history of diabetes mellitus, systolic blood pressure (SBP), and diastolic blood pressure (DBP), except for the variable being stratified. The size of the square represents the sample size of each subgroup. P for interaction was calculated to test the heterogeneity of associations across subgroups. TyG, triglyceride-glucose index; TyG-WHR, triglyceride-glucose-waist-to-hip ratio index; OR, odds ratio; CI, confidence interval; BMI, body mass index; SBP, systolic blood pressure; DBP, diastolic blood pressure.

To further evaluate the heterogeneity of the associations of TyG and TyG-WHR, subgroup analyses were conducted across different demographic and clinical characteristics, and the pooled results of the development set and validation set are presented in **Figure 2**.

In the development set (**Figure 2A**), the TyG index showed no significant association with CP risk among females (OR=1.176, P=0.141), individuals aged ≥ 60 years (OR=1.062, P=0.717), or those with hypertension (OR=1.114, P=0.310) or diabetes mellitus (OR=1.234, P=0.109), and a significant interaction with sex was observed (P-for-interaction < 0.001). In contrast, TyG-WHR demonstrated a significant positive association across all subgroups analyzed (all P < 0.05). Significant interactions were observed between TyG-WHR and sex, history of hypertension, and history of diabetes mellitus.

In the independent temporal validation set (**Figure 2C**), TyG-WHR maintained robust significance across all subgroups and exhibited statistically significant interactions with sex (P < 0.001), age (P < 0.001), and history of diabetes mellitus (P=0.002). TyG-WHR showed stronger associations among females, older adults (≥ 60 years), and individuals without diabetes.

### 3.4 Dose-response relationship between TyG-WHR/TyG index and prevalent carotid plaque

**Figure 3:**
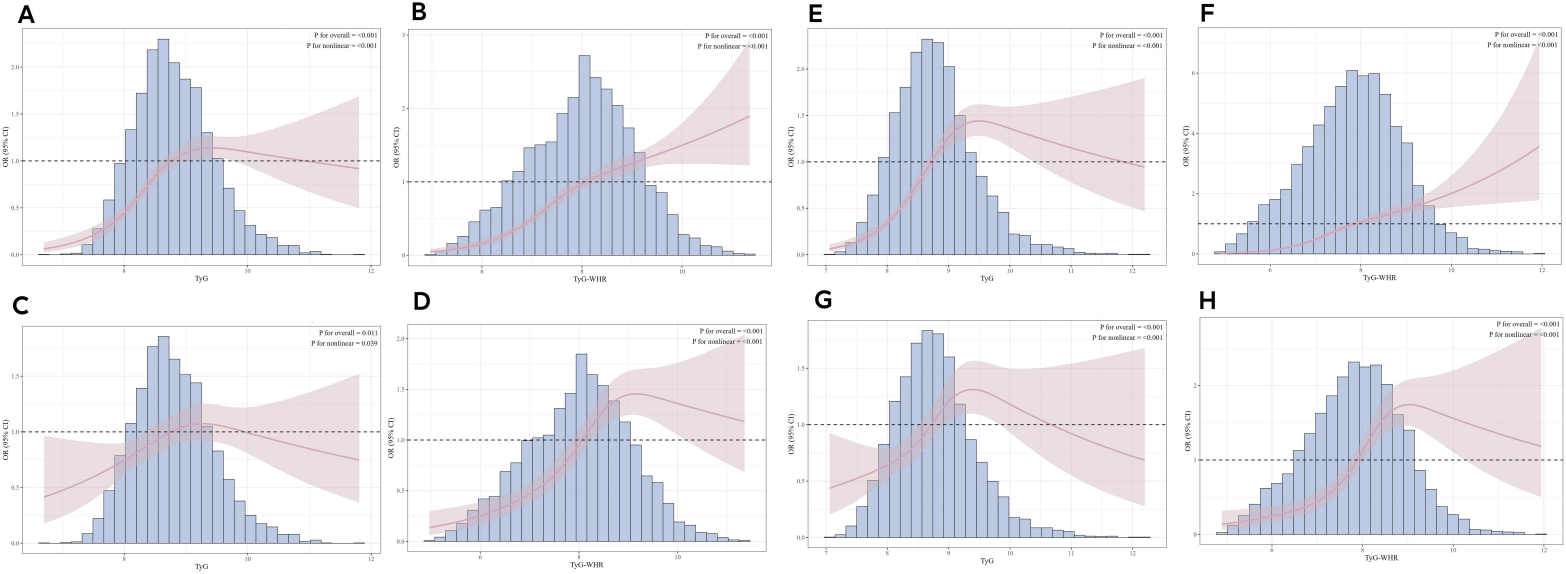
Dose-response relationships between TyG/TyG-WHR and carotid plaque analyzed by restricted cubic splines. **(A)** Unadjusted TyG in the development cohort. **(B)** Unadjusted TyG-WHR in the development cohort. **(C)** Adjusted TyG in the development cohort. **(D)** Adjusted TyG-WHR in the development cohort. **(E)** Unadjusted TyG in the temporal validation cohort. **(F)** Unadjusted TyG-WHR in the temporal validation cohort. **(G)** Adjusted TyG in the temporal validation cohort. **(H)** Adjusted TyG-WHR in the temporal validation cohort. Adjusted models were controlled for sex, age, body mass index (BMI), history of hypertension, history of diabetes mellitus, systolic blood pressure (SBP), and diastolic blood pressure (DBP). The solid line represents the odds ratio (OR), and the shaded area indicates the 95% confidence interval. The reference value (OR=1) is set at the median of the index distribution. P for non-linearity tests the deviation from a linear relationship. TyG, triglyceride-glucose index; TyG-WHR, triglyceride-glucose-waist-to-hip ratio index; RCS, restricted cubic spline; OR, odds ratio; CI, confidence interval.

In the development set, the TyG index exhibited a nonlinear association with CP risk (P-for-nonlinearity=0.015; **Figure 3**), and the TyG-WHR index also demonstrated a significant nonlinear association (P-for-nonlinearity < 0.001; **Figure 3**), with a progressively increasing risk of prevalent carotid plaque as the index level rose.

This pattern was essentially replicated in the independent temporal validation set. After full adjustment, the association between the TyG index and prevalent carotid plaque also deviated from linearity (P-for-nonlinearity=0.002; **Figure 3**), while the TyG-WHR index exhibited a nonlinear dose-response relationship (P-for-nonlinearity < 0.001, **Figure 3**), with the risk curve rising more steeply at higher index levels.

### 3.5 Discriminatory performance of TyG-WHR and TyG index for prevalent carotid plaque

**Figure 4:**
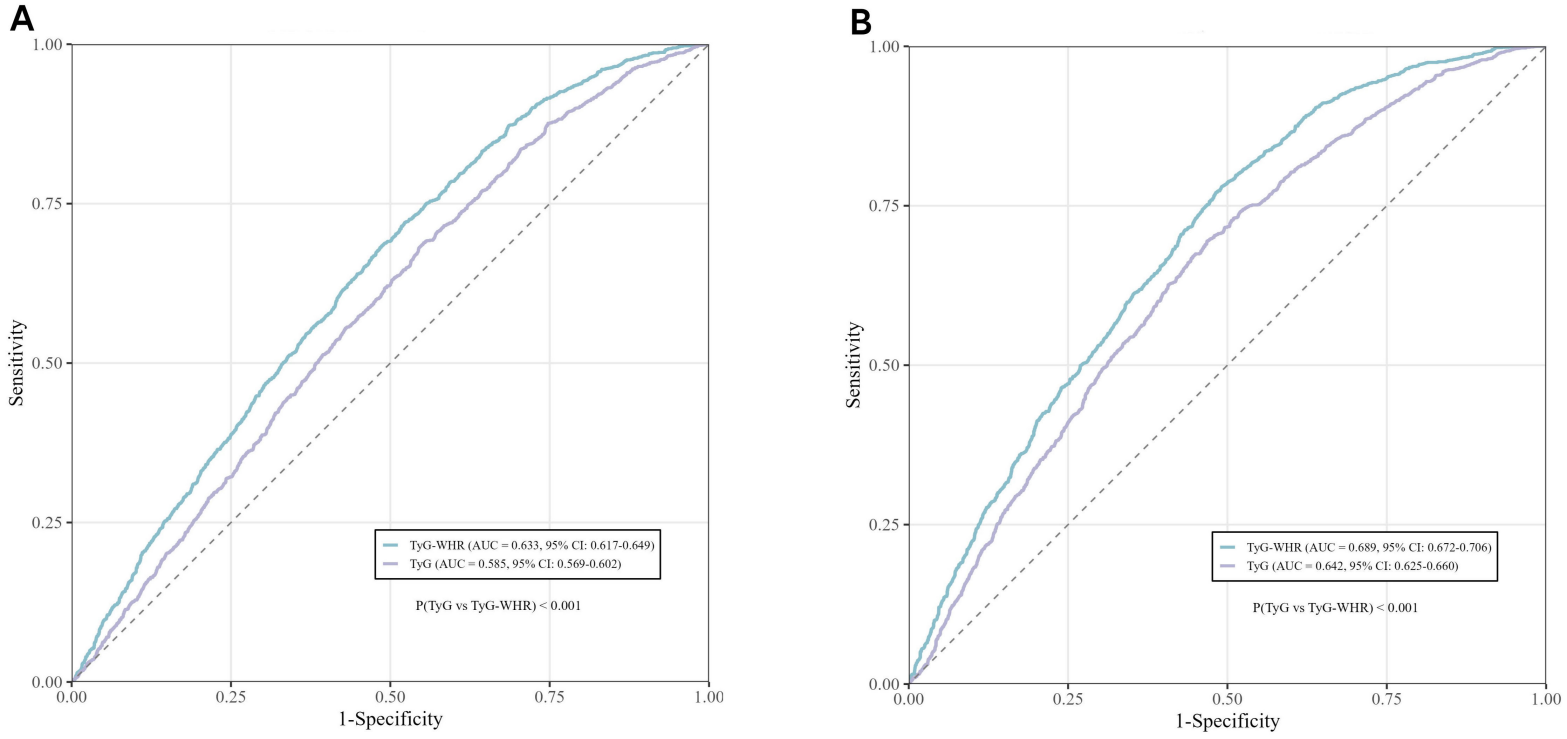
Discriminative performance of TyG and TyG-WHR for carotid plaque in the development cohort and temporal validation cohort. TyG, triglyceride-glucose index; TyG-WHR, triglyceride-glucose-waist-to-hip ratio index; AUC, area under the receiver operating characteristic curve; CI, confidence interval.

As shown in **Figure 4**, in the development set, the AUC of TyG-WHR was 0.633 (95% CI: 0.617-0.649), which was significantly superior to the 0.585 (95% CI: 0.569-0.602) of the TyG index. At the optimal cutoff value (7.84), TyG-WHR achieved a sensitivity of 72.05%, a specificity of 47.75%, and a maximum Youden’s index of 0.198.

As shown in **Figure 4**, in the independent temporal validation set, the AUC of TyG-WHR was 0.689 (95% CI: 0.672-0.706), compared with 0.642 (95% CI: 0.625-0.660) for the TyG index. At its optimal cutoff value of 7.475, TyG-WHR achieved a sensitivity of 78.01% and a maximum Youden’s index of 0.289.

### 3.6 Feature selection

To construct a robust and interpretable diagnostic model, a systematic two-step feature screening process was implemented.

**Figure 5:**
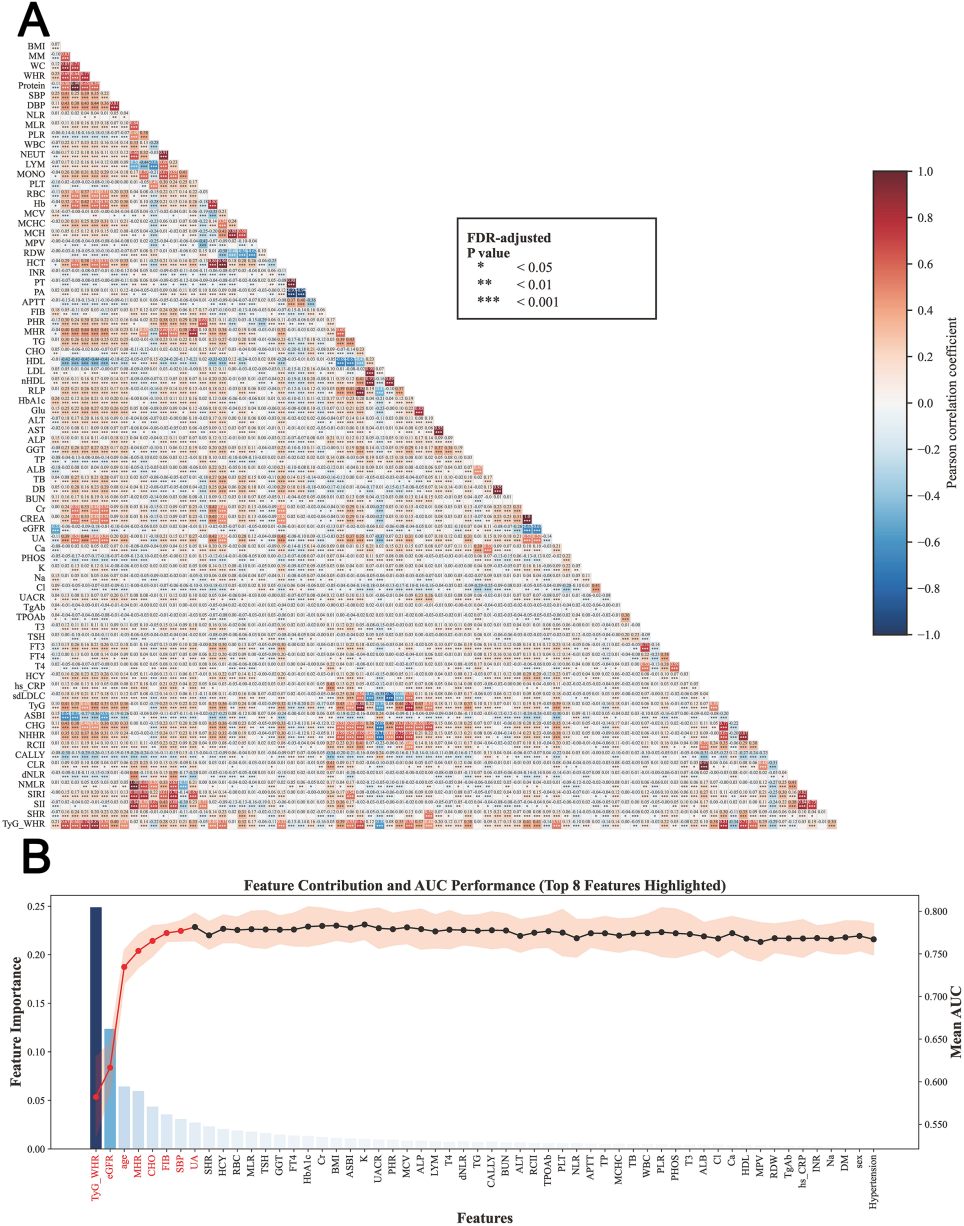
Correlation heatmap of selected variables and feature selection process based on Recursive Feature Elimination (RFE). **(A)** Correlation Heatmap of Selected Variables. **(B)** Recursive Feature Elimination (RFE) Process and Feature Contribution. RFE, Recursive Feature Elimination.

First, collinearity analysis was performed on all 84 candidate variables to identify and remove highly correlated variables, thereby reducing the risk of multicollinearity. Based on the correlation analysis, for each pair of highly correlated variables, the variable with greater clinical relevance to the outcome was retained, ultimately yielding 56 relatively independent variables (**Figure 5**).

Subsequently, based on these 56 variables, core feature selection was conducted using RFE combined with a random forest (RF) classifier. The performance of different feature subsets (measured by AUC) was evaluated via five-fold cross-validation to determine the optimal number. **Figure 5** illustrates the RFE process, demonstrating that model performance peaked when the number of features was reduced to eight.

Ultimately, eight core predictors were selected: age, estimated glomerular filtration rate (eGFR), TyG-WHR, systolic blood pressure (SBP), fibrinogen (FIB), uric acid (UA), monocyte-to-high-density lipoprotein cholesterol ratio (MHR), and total cholesterol (CHO). The present study further performed multicollinearity testing on the selected indices, all of which met the criteria (**Supplementary Table 5**). This set of indices comprehensively encompasses key pathophysiological dimensions including demographics, metabolism, inflammation, coagulation, and renal function. To evaluate the generalization error of the complete modeling pipeline including feature selection, 10-fold nested cross-validation was performed (outer 10 folds for assessing generalization performance, inner 5 folds for RFE and hyperparameter tuning). The mean AUC was 0.791, largely consistent with the internal validation set AUC of 0.793 (difference = 0.002), indicating that the feature selection process did not induce substantive overfitting. Across the 10 folds, six variables—age, SBP, FIB, MHR, eGFR, and TyG-WHR—were stably selected in every fold, while the remaining two positions exhibited some competition among UA, CHO, HbA1c, and HCY; however, the overall generalization performance of the model remained stable (see **Supplementary Table 6** for details).

### 3.7 Model evaluation

Based on the eight selected core features, a logistic regression model was constructed on the training set as the primary model and presented in the form of a nomogram. As sensitivity analyses, six additional models-decision tree, random forest, XGBoost, LightGBM, support vector machine (SVM), and artificial neural network (ANN)-were constructed for comparison. All models underwent hyperparameter optimization via grid search combined with five-fold cross-validation (optimal parameters are detailed in **Supplementary Table 7**), and were comprehensively evaluated on the independent internal validation set and temporal validation set. The ROC curves, calibration curves, and clinical decision curves of each model on the training set are provided in **Supplementary Figure 1**.

**Figure 6:**
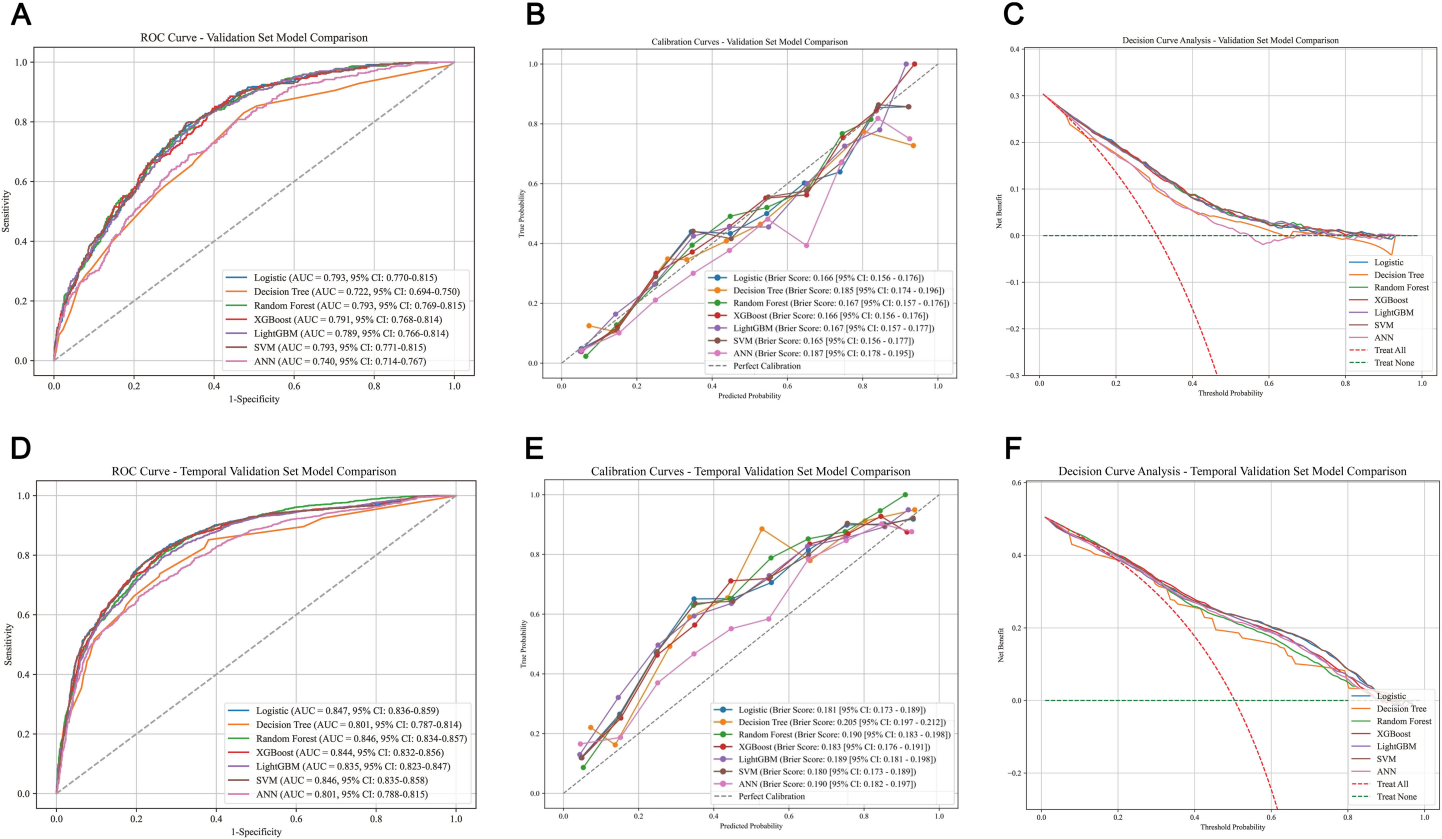
Assessment of model performance on the validation set and temporal validation set. **(A)** Receiver Operating Characteristic (ROC) curves on the Validation Cohort. **(B)**Calibration curves on the Validation Cohort. **(C)** Decision Curve Analysis (DCA) on the Validation Cohort. **(D)** Receiver Operating Characteristic (ROC) curves on the Temporal validation set. **(E)** Calibration curves on the Temporal validation set. **(F)** Decision Curve Analysis (DCA) on the Temporal validation set. AUC, area under the curve; CI, confidence interval; XGBoost,eXtreme Gradient Boosting; LightGBM,Light Gradient Boosting Machine; SVM, support vector machine; ANN, artificial neural network.

Across the three sets, the present study uniformly adopted 0.5 as the classification threshold. On the internal validation set, all models demonstrated robust discriminatory ability, with AUROC values ranging from 0.74 to 0.79 (**Table 3**). Among them, logistic regression, SVM, XGBoost, and LightGBM exhibited comparable performance (AUROC=0.79 for all). **Figure 6A** presents the detailed evaluation results of the logistic regression model on the internal validation set: its AUROC was 0.793 (95% CI: 0.770-0.815); the calibration curve shown in **Figure 6** indicated good calibration (Brier Score: 0.167); and decision curve analysis (DCA; **Figure 6**) demonstrated that the model provided net clinical benefit across a clinically relevant range of thresholds. In the temporal validation set (**Figure 6D**), the logistic regression model achieved an AUROC of 0.85, with the calibration curve closely approximating the ideal diagonal line (Brier Score: 0.182), and DCA likewise demonstrated robust net benefit.

**Table 3:** Performance of machine learning models for carotid plaque stratification on the training, internal validation, and temporal validation sets.

Model	AUROC (95% CI)	Accuracy	Precision	Sensitivity	Specificity	F1 Score	Kappa	Youden’s J	PPV	NPV
Training set										
Logistic Regression	0.79(0.77-0.81)	0.75	0.63	0.43	0.89	0.51	0.35	0.32	0.63	0.78
Decision Tree	0.77(0.75-0.79)	0.75	0.69	0.34	0.93	0.45	0.31	0.27	0.69	0.76
Random Forest	0.81(0.80-0.83)	0.77	0.70	0.42	0.92	0.53	0.38	0.34	0.70	0.78
XGBoost	0.83(0.81-0.84)	0.77	0.73	0.44	0.92	0.55	0.41	0.37	0.73	0.79
LightGBM	0.87(0.85-0.88)	0.81	0.77	0.54	0.93	0.64	0.52	0.47	0.77	0.82
SVM	0.79(0.77-0.81)	0.75	0.63	0.44	0.89	0.52	0.36	0.33	0.63	0.78
ANN	0.75(0.73-0.77)	0.71	0.54	0.51	0.80	0.52	0.32	0.31	0.54	0.78
Validation set										
Logistic Regression	0.79(0.77-0.81)	0.74	0.62	0.42	0.88	0.50	0.33	0.30	0.62	0.77
Decision Tree	0.72(0.69-0.75)	0.72	0.62	0.29	0.92	0.39	0.24	0.21	0.62	0.74
Random Forest	0.79(0.77-0.82)	0.74	0.63	0.38	0.90	0.47	0.31	0.28	0.63	0.76
XGBoost	0.79(0.77-0.81)	0.74	0.63	0.40	0.90	0.49	0.33	0.30	0.63	0.77
LightGBM	0.79(0.77-0.81)	0.73	0.60	0.42	0.88	0.49	0.32	0.29	0.60	0.77
SVM	0.79(0.77-0.81)	0.74	0.62	0.42	0.88	0.51	0.34	0.31	0.62	0.77
ANN	0.74(0.71-0.77)	0.71	0.53	0.50	0.80	0.51	0.30	0.30	0.53	0.78
Temporal validation set										
Logistic Regression	0.85(0.84-0.86)	0.73	0.87	0.55	0.91	0.67	0.46	0.46	0.87	0.66
Decision Tree	0.80(0.79-0.81)	0.68	0.86	0.44	0.92	0.59	0.37	0.37	0.86	0.62
Random Forest	0.85(0.83-0.86)	0.71	0.87	0.51	0.92	0.64	0.43	0.43	0.87	0.64
XGBoost	0.84(0.83-0.86)	0.72	0.86	0.55	0.91	0.67	0.45	0.45	0.86	0.66
LightGBM	0.84(0.82-0.85)	0.72	0.85	0.55	0.90	0.67	0.45	0.45	0.85	0.66
SVM	0.85(0.84-0.86)	0.73	0.86	0.55	0.91	0.68	0.46	0.46	0.86	0.66
ANN	0.80(0.79-0.81)	0.71	0.80	0.58	0.85	0.67	0.43	0.43	0.80	0.66

AUROC, Area Under the Receiver Operating Characteristic Curve; CI, Confidence Interval; PPV, Positive Predictive Value; NPV, Negative Predictive Value; Logistic, Logistic Regression; Decision Tree, Decision Tree; Random Forest, Random Forest; XGBoost, Extreme Gradient Boosting; LightGBM, Light Gradient Boosting Machine; SVM, Support Vector Machine; ANN, Artificial Neural Network.

To further compare performance differences among models, DeLong’s test was applied to compare the AUCs of all models in the training set, internal validation set, and temporal validation set in a pairwise manner. As shown in **Supplementary Table 8**, DeLong’s test revealed no statistically significant difference between logistic regression and SVM in either the internal validation set or the temporal validation set (P > 0.05). With respect to the Brier Score, SVM achieved values of 0.166 and 0.181 in the internal and temporal validation sets respectively, compared with 0.167 and 0.182 for logistic regression; the differences between them were minimal (Δ=0.001 only) and clinically inconsequential. Given that logistic regression and SVM were at the same level in terms of both discriminatory performance and calibration, and that logistic regression can be condensed into a nomogram for paper-and-pencil use without reliance on complex computation, thereby enabling convenient deployment in primary care settings-in accordance with Occam’s razor, when performance is equivalent, the simpler and more transparent model should be preferred-logistic regression was therefore selected as the final model in this study. Taking into account the intended role of this nomogram as a preliminary screening tool, a high-sensitivity screening cutoff was determined on the training set with the objective of achieving a sensitivity ≥ 0.80. At this cutoff (predicted probability=0.25), the performance in the temporal validation set was as follows: sensitivity=0.81, specificity=0.75, positive predictive value (PPV)=0.49, negative predictive value (NPV)=0.89, and F1 score=0.61. This cutoff is recommended to minimize missed diagnoses. For the logistic regression model, the Hosmer-Lemeshow goodness-of-fit test yielded the following results: training set χ^2^=13.54 (P=0.095), internal validation set χ^2^=14.83 (P=0.063), and temporal validation set χ^2^=14.11 (P=0.083). The P-values for all three datasets were > 0.05, suggesting no significant discrepancy between the predicted probabilities and the observed probabilities of the nomogram. The Brier Score for the temporal validation set was 0.182 (95% CI: 0.174-0.190), indicating acceptable model calibration.

### 3.8 Nomogram

**Figure 7:**
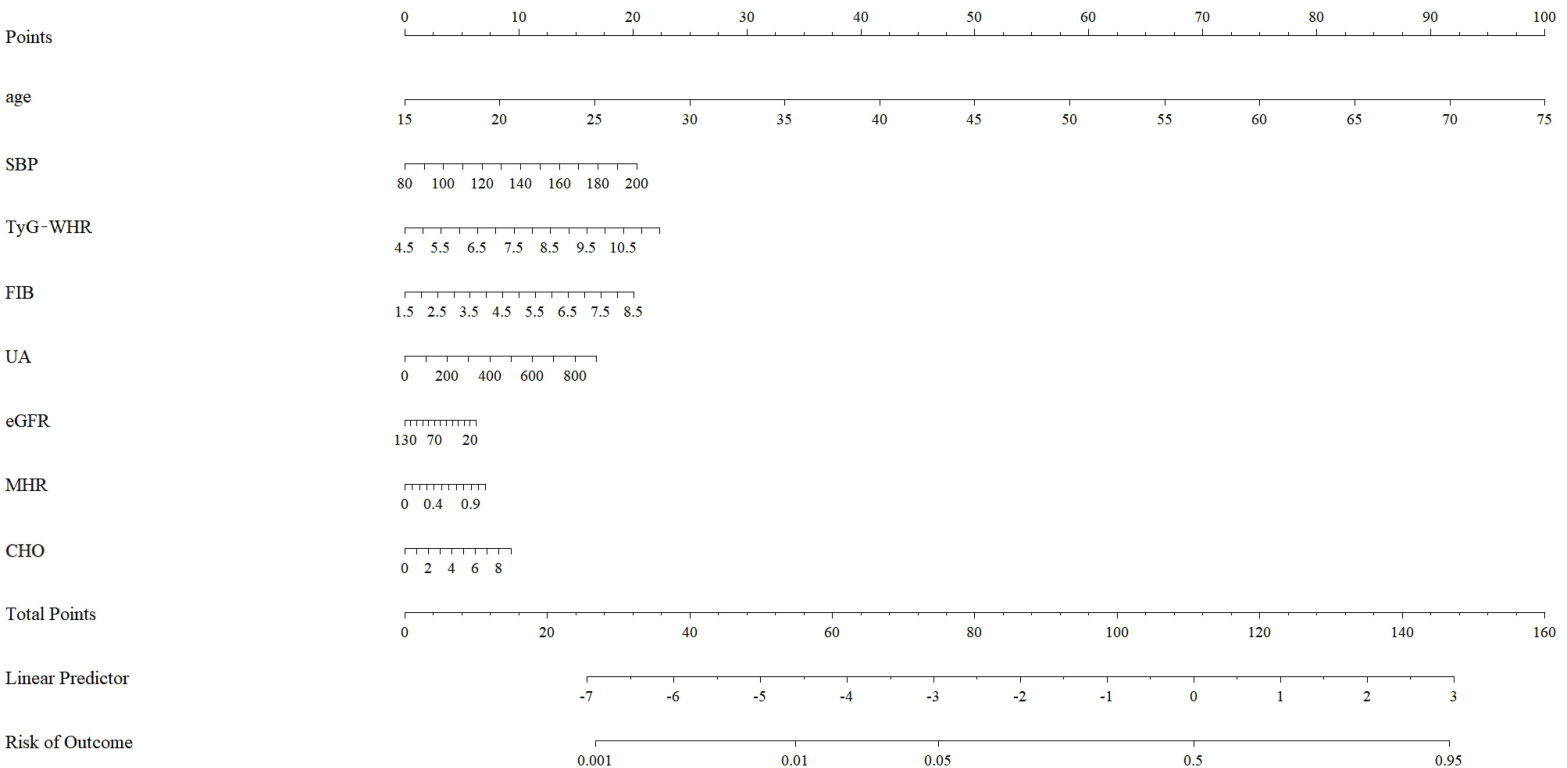
Nomogram for estimating the probability of prevalent carotid plaque. **(A)** Nomogram for estimating the probability of prevalent carotid plaque based on eight routinely available variables. To use the nomogram, locate a variable’s value on its corresponding axis, draw a vertical line upward to the “Points” axis to obtain the score for that variable, repeat for all eight variables, sum all scores to obtain the total points, and then read the corresponding estimated probability from the “Risk of Outcome” axis. The predicted probability reflects the likelihood of currently having carotid plaque, not the risk of developing future plaque. SBP, systolic blood pressure (mmHg); TyG-WHR, triglyceride-glucose-waist-to-hip ratio; FIB, fibrinogen (g/L); UA, uric acid (μmol/L); eGFR, estimated glomerular filtration rate (mL/min/1.73 m^2^); MHR, monocyte-to-HDL cholesterol ratio; CHO, total cholesterol (mmol/L).

**Figure 7** presents the nomogram based on the logistic regression model. This nomogram converts the regression coefficients of the eight variables into intuitive scoring scales: clinical users can sum the points corresponding to each variable to obtain a total score, and then read the corresponding probability of prevalent carotid plaque from the probability axis at the bottom of the nomogram. The multivariable logistic regression coefficients corresponding to the nomogram are presented in **Table 4**. All eight variables were significantly associated with prevalent carotid plaque (all P < 0.05). Each 1-year increase in age was associated with a 14.7% increase in the risk of prevalent plaque (OR = 1.147, 95% CI: 1.130–1.163); each 1-unit increase in MHR was associated with a 70.0% increase in risk (OR = 1.700, 95% CI: 1.046–2.765); and each 1-unit increase in TyG-WHR was associated with a 30.0% increase in risk (OR = 1.300, 95% CI: 1.161–1.456). To further facilitate the practical application of the nomogram in health examination settings, **Supplementary Table 9** provides the explicit formulas for converting the raw measurement values of the eight variables into nomogram points: Points = b_0_ + x^1^ × Value, where the intercept (b_0_) and slope (x^1^) are specific to each variable. The applicable value range (Range-Min to Range-Max) for each variable, corresponding to the range observed in the development cohort, is also listed. By summing the individual point scores across all variables to obtain a total score, clinicians can directly read the corresponding estimated probability of prevalent carotid plaque from the “Risk of Outcome” axis at the bottom of the nomogram, without the need to manually draw vertical lines on the chart, thereby enabling rapid batch scoring. In addition, for digital systems requiring direct computation of individual predicted probabilities, the linear predictor (LP) derived from the multivariable logistic regression model can be used: LP = −13.895 + 0.137 × age + 0.014 × SBP + 0.263 × TyG-WHR + 0.236 × FIB + 0.002 × UA − 0.004 × eGFR + 0.531 × MHR + 0.085 × CHO, and the individual predicted probability of prevalent carotid plaque is calculated as P = 1/[1 + exp(−LP)]. These two approaches are mathematically equivalent: the nomogram-based scoring system is suitable for rapid consultation of printed reports, whereas the LP formula facilitates automated risk computation and batch screening in electronic health examination systems.

**Table 4:** Multivariable logistic regression coefficients for the nomogram.

Variable	Beta	SE	Wald	OR	Lower-CI	Upper-CI	p-value
(Intercept)	-13.895	0.865	-16.069	–	–	–	<0.001
age	0.137	0.007	18.657	1.147	1.130	1.163	<0.001
SBP	0.014	0.003	4.794	1.014	1.008	1.020	<0.001
TyG-WHR	0.263	0.058	4.555	1.300	1.161	1.456	<0.001
FIB	0.236	0.075	3.126	1.266	1.092	1.467	0.002
UA	0.002	0.001	2.594	1.002	1.000	1.003	0.009
eGFR	-0.004	0.002	-2.286	0.996	0.993	0.999	0.022
MHR	0.531	0.248	2.141	1.700	1.046	2.765	0.032
CHO	0.085	0.038	2.257	1.089	1.011	1.173	0.025

β, regression coefficient; SE, standard error; OR, odds ratio; CI, confidence interval; SBP, systolic blood pressure; TyG-WHR, triglyceride-glucose-waist-to-hip ratio; FIB, fibrinogen; UA, uric acid; eGFR, estimated glomerular filtration rate; MHR, monocyte-to-HDL cholesterol ratio; CHO, total cholesterol.

### 3.9 Incremental value of TyG-WHR

A base model was constructed using traditional clinical variables including age, sex, BMI, SBP, diastolic blood pressure (DBP), history of hypertension, and history of diabetes mellitus to evaluate the incremental value of TyG-WHR (**Table 5**).

**Table 5:** Incremental value of TyG-WHR over the basic model for carotid plaque screening.

Model.	C statistic estimate (95% CI)	C statistic p value	NRI estimate (95% CI)	NRI p value	IDI estimate (95% CI)	IDI p value	LR χ2	LR p value
Validation set								
Basic model	0.657 (0.627-0.688)	<0.001	Reference		Reference		Reference	
Basic model + TyG-WHR	0.667 (0.639-0.696)	<0.001	0.2105 (0.0987-0.3261)	<0.001	0.0085 (0.0032-0.0141)	<0.001	37.195	<0.001
Temporal validation set								
Basic model	0.692 (0.675-0.709)	<0.001	Reference		Reference		Reference	
Basic model + TyG-WHR	0.745 (0.730-0.760)	<0.001	0.4724 (0.4159-0.5322)	<0.001	0.0247 (0.0219-0.0279)	<0.001	37.195	<0.001

Basic model included age, sex, BMI; systolic blood pressure (SBP); diastolic blood pressure (DBP); history of hypertension, and history of diabetes mellitus. C statistic, area under the receiver operating characteristic curve; NRI, net reclassification improvement; IDI, integrated discrimination improvement; LR χ^2^, likelihood ratio chi-square statistic.

In the internal validation set, the addition of TyG-WHR increased the C-statistic from 0.657 (95% CI: 0.627-0.688) to 0.667 (95% CI: 0.639-0.696); the NRI was 0.211 (95% CI: 0.099-0.326, P < 0.001), the integrated discrimination improvement (IDI) was 0.009 (95% CI: 0.003-0.014, P < 0.001), and the likelihood ratio test χ^2^=37.20 (P < 0.001). In the temporal validation set, the C-statistic increased from 0.692 (95% CI: 0.675-0.709) to 0.745 (95% CI: 0.730-0.760); the NRI was 0.472 (95% CI: 0.416-0.532, P < 0.001), the IDI was 0.025 (95% CI: 0.022-0.028, P < 0.001), and the likelihood ratio test χ^2^=37.20 (P < 0.001).

In both datasets, TyG-WHR achieved statistical significance across all incremental value metrics; in the temporal validation set, the increase in the C-statistic reached 0.053 and the NRI reached 0.472, suggesting that TyG-WHR provides a clear and reproducible incremental contribution to carotid plaque risk screening beyond traditional clinical variables.

### 3.10 Model performance in different subgroups

To assess the applicable boundaries of the nomogram across subgroups, stratified subgroup analyses (exploratory analyses) were conducted by sex, BMI, history of diabetes mellitus, history of hypertension, and age. All 95% CIs of the AUCs were adjusted for multiple comparisons using the Bonferroni method; detailed results are presented in **Table 6**.

**Table 6:** Performance evaluation of the Logistic Regression(LR) model in each subgroup.

Model	N	Events	AUC (95% CI, Bonferroni)	Sensitivity	Specificity	Youden’s J	PPV	NPV	F1 Score	Kappa
Training set										
Male	2324	819	0.77 (0.73-0.79)	0.41	0.87	0.29	0.64	0.73	0.50	0.31
Female	1172	266	0.83 (0.79-0.87)	0.48	0.91	0.40	0.62	0.86	0.55	0.43
BMI<24	1254	289	0.81 (0.77-0.84)	0.35	0.92	0.27	0.56	0.82	0.43	0.31
BMI≥24	2242	796	0.78 (0.75-0.80)	0.46	0.87	0.33	0.65	0.75	0.54	0.35
Diabetes mellitus=0	3000	838	0.79 (0.76-0.81)	0.38	0.90	0.29	0.60	0.79	0.47	0.32
Diabetes mellitus=1	496	247	0.74 (0.68-0.80)	0.60	0.75	0.35	0.70	0.65	0.65	0.35
Hypertension=0	2503	680	0.80 (0.78-0.83)	0.36	0.92	0.29	0.63	0.80	0.46	0.33
Hypertension=1	993	405	0.75 (0.70-0.79)	0.54	0.78	0.32	0.63	0.71	0.58	0.33
age<60	3073	781	0.75 (0.73-0.78)	0.23	0.93	0.16	0.52	0.78	0.32	0.19
age≥60	423	304	0.65 (0.57-0.73)	0.95	0.09	0.04	0.73	0.42	0.82	0.06
Validation set										
Male	959	336	0.76 (0.71-0.80)	0.40	0.86	0.27	0.62	0.73	0.49	0.29
Female	537	128	0.85 (0.80-0.90)	0.47	0.91	0.38	0.62	0.85	0.53	0.41
BMI<24	571	127	0.85 (0.80-0.89)	0.39	0.94	0.33	0.64	0.84	0.48	0.38
BMI≥24	925	337	0.75 (0.70-0.79)	0.44	0.84	0.27	0.61	0.72	0.51	0.29
Diabetes mellitus=0	1289	351	0.79 (0.75-0.83)	0.38	0.90	0.29	0.60	0.80	0.47	0.32
Diabetes mellitus=1	207	113	0.70 (0.60-0.80)	0.54	0.67	0.21	0.66	0.55	0.60	0.21
Hypertension=0	1044	277	0.81 (0.77-0.84)	0.35	0.91	0.26	0.59	0.79	0.44	0.30
Hypertension=1	452	187	0.74 (0.67-0.80)	0.53	0.79	0.32	0.64	0.71	0.58	0.33
age<60	1307	337	0.77 (0.73-0.80)	0.22	0.93	0.15	0.52	0.77	0.31	0.19
age≥60	189	127	0.68 (0.58-0.78)	0.95	0.13	0.08	0.69	0.57	0.80	0.10
Temporal validation set										
Male	2110	1017	0.82 (0.79-0.84)	0.42	0.89	0.31	0.79	0.62	0.54	0.32
Female	1702	925	0.88 (0.85-0.90)	0.69	0.94	0.63	0.93	0.72	0.79	0.62
BMI<24	1503	566	0.89 (0.86-0.91)	0.56	0.94	0.50	0.85	0.78	0.68	0.54
BMI≥24	2309	1376	0.81 (0.78-0.83)	0.54	0.89	0.43	0.87	0.57	0.67	0.39
Diabetes mellitus=0	3123	1420	0.84 (0.82-0.86)	0.50	0.93	0.42	0.85	0.69	0.63	0.44
Diabetes mellitus=1	689	522	0.81 (0.75-0.86)	0.69	0.78	0.47	0.91	0.44	0.78	0.37
Hypertension=0	2760	1068	0.83 (0.81-0.85)	0.41	0.95	0.36	0.83	0.72	0.55	0.39
Hypertension=1	1052	874	0.71 (0.64-0.76)	0.71	0.61	0.33	0.90	0.30	0.80	0.23
age<60	2781	1045	0.79 (0.77-0.81)	0.17	0.97	0.15	0.79	0.66	0.28	0.17
age≥60	1031	897	0.66 (0.58-0.74)	0.98	0.16	0.14	0.89	0.57	0.93	0.20

AUC, area under the receiver operating characteristic curve; CI, confidence interval; PPV, positive predictive value; NPV, negative predictive value; BMI, body mass index. All AUC 95% CIs were adjusted using the Bonferroni method for multiple comparisons across subgroups. These subgroup analyses were exploratory.

Across the three datasets, the nomogram performed relatively well in females (internal validation set AUC=0.85; temporal validation set AUC=0.88), individuals with BMI < 24 kg/m^2^ (internal validation set AUC=0.85; temporal validation set AUC=0.89), and the subgroup without diabetes mellitus. The AUCs for the age < 60 years subgroup were 0.75, 0.77, and 0.79 in the training set, internal validation set, and temporal validation set, respectively.

Two points merit particular attention. First, the AUCs for the age ≥ 60 years subgroup were only 0.65, 0.68, and 0.66 across the three datasets, approaching random level and therefore lacking practical screening value. Second, the AUCs for the hypertension subgroup were 0.75, 0.74, and 0.71, respectively, all falling below the acceptable screening standard. Accordingly, the applicable population of this nomogram is recommended to be restricted to individuals aged < 60 years without hypertension.

## 4 Discussion

Leveraging a large health examination cohort, this study comprehensively and systematically evaluated the association between the novel composite index TyG-WHR and prevalent carotid plaque, and concurrently constructed a cross-sectional risk screening nomogram. After thorough adjustment for confounding factors, the results demonstrated that TyG-WHR is an independent risk indicator for prevalent carotid plaque, with discriminatory performance significantly superior to that of the conventional TyG index-a finding that was corroborated in the temporal validation set. By integrating multidimensional routine indices, we developed a logistic regression-based nomogram that exhibited moderate discriminatory ability and good calibration in both internal validation and temporal validation.

The finding of this study that TyG-WHR outperforms the TyG index in discriminating prevalent carotid plaque has a clear pathophysiological basis. The TyG index is a reliable surrogate marker for assessing IR, which directly promotes the initiation and progression of atherosclerosis by fostering endothelial dysfunction (28), oxidative stress, and chronic inflammation (29, 30). However, the TyG index fails to capture the critical dimension of fat distribution. TyG-WHR simultaneously integrates the two key pathological processes of IR and adverse fat distribution, thereby enabling a more comprehensive assessment of metabolic atherosclerotic risk (31–34). The present study demonstrated that TyG-WHR exhibited a stronger association with carotid plaque among non-diabetic individuals, suggesting that this index may more sensitively identify individuals with pre-existing vascular damage before the manifestation of clinical hyperglycemia, thus aiding in the detection of high-risk populations who warrant further imaging evaluation.

The incremental value of TyG-WHR over the base model was validated in both the internal validation set and the temporal validation set. When TyG-WHR was added to a base model comprising only traditional clinical variables (age, sex, BMI, blood pressure, history of hypertension, and history of diabetes mellitus), the NRI and likelihood ratio test were significant in both datasets, and the IDI was positive and significant in both. The C-statistic increased in both the internal and temporal validation sets, indicating that TyG-WHR carries independent information not captured by traditional clinical variables. IR and abnormal fat distribution have been established as independent pathological mechanisms of atherosclerosis; TyG-WHR, as a composite surrogate index for both, provides discriminatory and stratifying value for prevalent carotid plaque screening beyond that offered by traditional risk factors. For reference, Pavluk et al. reported an incremental NRI of 0.48 (P = 0.017) for AI-ECG age in predicting carotid plaque volume in a small prospective cohort (n = 101) (35), which is comparable in magnitude to the NRI of 0.472 observed for TyG-WHR in the temporal validation set of the present study; however, the current study involved a substantially larger sample and employed a clinically more accessible outcome—the presence versus absence of plaque—rather than quantitative plaque volume.

With respect to metabolic risk indicators for carotid plaque, He et al., using longitudinal data from 10,407 participants in the Dalian Health Management Cohort (36), demonstrated that the TyG index and its derivative indices (TyG-BMI, TyG-WC, TyG-WHtR) were all significantly associated with the risk of incident CP, with TyG-WC and TyG-WHtR showing superior discriminatory performance compared with the TyG index alone. This finding supports the rationale of combining central obesity measures with the TyG index; however, the waist-to-hip ratio (WHR) dimension was not within the scope of that comparison. In the present study, TyG-WHR also outperformed the TyG index in cross-sectional screening, providing complementary evidence for this composite strategy from a different study design (cross-sectional vs. longitudinal) and a different obesity metric (WHR vs. WC/WHtR). Jang et al., in a cross-sectional study of an asymptomatic Korean population (n = 801) (37), reported a CP prevalence of 22.1% and identified age, HbA1c, male sex, hypertension, and multiple plaques as independent predictors of high-risk plaque; the spectrum of traditional risk factors identified in that study is consistent with the present findings, although that work focused on plaque morphological characteristics rather than providing an actionable risk stratification tool in the form of a nomogram. In addition, with regard to CP screening modeling using routine indices, Bin et al. developed an XGBoost model (AUC = 0.808) based on 4,659 health examination records (38), and Weng et al. constructed a LightGBM model (AUC = 0.972) using data from 5,211 individuals (39), both demonstrating the feasibility of CP screening using routine health examination indices. Compared with the aforementioned studies, the distinguishing features of the present work lie in: incorporating TyG-WHR (which integrates insulin resistance with fat distribution assessed by WHR) into the screening model and validating its incremental value beyond traditional clinical variables; presenting the model in the form of a nomogram, thereby lowering the barrier to deployment in primary care settings; and preliminarily delineating the applicable and non-applicable population boundaries of the model through subgroup analyses.

The other indices in the model also point to core aspects of atherosclerosis: age represents cumulative damage and the temporal dimension; TyG-WHR integrates visceral adiposity and IR, which are core drivers of metabolic inflammation; SBP reflects the mechanical stress exerted by hemodynamic load on the vessel wall; FIB is a dual marker of the coagulation system and the acute-phase inflammatory response (40–42); MHR uniquely links inflammatory cell activity with reverse cholesterol transport function (43); UA can inhibit the production of nitric oxide (NO)-a critical vasodilator and anti-atherosclerotic molecule-in the vascular endothelium, leading to impaired vasodilation and increased permeability, thereby creating conditions conducive to lipid infiltration and inflammatory cell recruitment (44, 45); CHO represents the classical lipid risk (45–49); and eGFR assesses the functional status of the kidney as a metabolic end-organ, whose decline is itself a marker of widespread vascular damage and may exacerbate hyperuricemia by affecting UA excretion (50).

Logistic regression, as a linear model, inherently carries a low risk of overfitting. In the present study, the final model contained only eight variables, and the ratio of events (1,085 cases) to the number of variables (EPV≈136) far exceeded the conventional recommendation (EPV ≥ 10-20), indicating that the model possesses adequate statistical power. At the same time, although the linearity assumption of logistic regression may not fully capture complex interaction effects among variables, the stability conferred by its simplicity is often preferable in clinical applications-particularly given that, in this study, more complex nonlinear models (e.g., SVM, random forest, XGBoost) did not demonstrate superior generalization ability over logistic regression on the validation sets.

The nomogram developed in this study offers a degree of convenience for clinical translation: all input variables are obtainable from routine health examinations without incurring additional costs. In community hospitals or health examination centers, this nomogram can serve as a preliminary screening tool prior to carotid ultrasound examination, rapidly identifying individuals with a higher probability of prevalent plaque while intuitively displaying the main risk drivers to guide targeted interventions. Considering the principle of prioritizing the minimization of missed diagnoses in health examination screening, this study recommends adopting a high-sensitivity cutoff (0.25), at which the sensitivity is 0.81, the specificity is 0.75, and the NPV reaches 0.89, thereby facilitating the effective exclusion of low-risk individuals and reducing unnecessary ultrasound referrals. DCA demonstrated that this nomogram yields positive net benefit across a wide range of thresholds, outperforming the default strategies of screening all or screening none, supporting its potential value as a clinical decision-support tool.

Subgroup analyses provided key information regarding the applicable boundaries of the model. The nomogram demonstrated relatively better discriminatory ability in women and normal-weight individuals, but its performance was notably insufficient in two high-prevalence subgroups. In the older adult subgroup (≥ 60 years), the prevalence of carotid plaque was as high as approximately 87%, resulting in a severe imbalance between positive and negative samples; the model struggled to further differentiate individual risk within this high-prevalence population, with its AUC approaching random level. The hypertension subgroup faced a similar challenge, where the superimposed effects of comorbidities and confounding factors limited the discriminatory capacity of routine indices. Moreover, the elevated PPV in high-prevalence subgroups—a mathematical inevitability as PPV automatically increases with prevalence—should not be taken as evidence of model validity; accordingly, the subgroup evaluation in this study consistently relied on the AUC as the core criterion. Based on these findings, this nomogram is currently not recommended for use in older adults aged ≥ 60 years or in patients with established hypertension. For these high-risk populations, dedicated screening tools incorporating more refined imaging features or novel biomarkers are needed in future research.

This study has the following limitations. First, this was a single-center, retrospective study; the temporal validation cohort was still derived from the same medical center and did not constitute true external validation. Moreover, the AUROC of the temporal validation set (0.85) was higher than that of the internal validation set (0.79), which may be attributable in part to differences in prevalence and demographic characteristics between the two cohorts rather than to an improvement in the model’s generalizability. The cross-institutional and cross-regional generalizability of the model remains to be validated in multicenter prospective cohorts. Second, the cross-sectional design dictates that this study can only assess associations between variables and existing carotid plaque, and cannot infer causality or predict future incident plaque; longitudinal follow-up studies are a necessary next step for evaluating its predictive value. Third, the health examination database did not capture medication history (e.g., statins, antihypertensive agents) or lifestyle information, which may introduce residual confounding. In particular, statins directly affect lipid levels and plaque stability, and the complete absence of this information constitutes an important limitation of the present model. Fourth, the discriminatory ability of the model in older adults (≥ 60 years) and hypertensive populations approached random level, explicitly limiting its applicability in these two populations. Furthermore, a lower screening threshold (sensitivity 0.81, specificity 0.75) had to be adopted to effectively reduce missed diagnoses in screening scenarios, at the cost of an elevated false-positive rate. Fifth, whether the actual clinical utility of this nomogram-i.e., its ability to improve screening efficiency or patient outcomes compared with current clinical judgment-has not been evaluated in prospective studies, and its real-world effectiveness remains to be validated.

## 5 Conclusion

This study systematically validated the independent discriminatory value of the TyG-WHR index for prevalent carotid plaque in the general population. Its discriminatory performance was superior to that of the conventional TyG index, and it provided significant and reproducible incremental information beyond traditional clinical variables. The nomogram constructed on this basis offers a convenient, low-cost auxiliary tool for carotid plaque risk stratification in large-scale health examination settings. This model is intended to identify individuals who may currently harbor plaque, rather than to predict future events.

## Data Availability

The raw data supporting the conclusions of this article will be made available by the authors, without undue reservation.

## Glossary

AUC: Area Under the Curve
AUROC: Area Under the Receiver Operating Characteristic Curve
ALB: Albumin
ALP: Alkaline Phosphatase
ALT: Alanine Aminotransferase
ANN: Artificial Neural Network
APTT: Activated Partial Thromboplastin Time
ASBI: Atherogenic Index of Plasma
AST: Aspartate Aminotransferase
BMI: Body Mass Index
BMR: Basal Metabolic Rate
BUN: Blood Urea Nitrogen
CALLY: C-Reactive Protein-Albumin-Lymphocyte Ratio
CEA: Carcinoembryonic Antigen
CHG: Cholesterol, high-density lipoprotein, and glucose index
CHO: Total Cholesterol
CI: Confidence Interval
CLR: C-Reactive Protein to Lymphocyte Ratio
CP: Carotid Plaque
Cr: Creatinine
CREA: Creatinine
DB: Direct Bilirubin
DBP: Diastolic Blood Pressure
dNLR: derived Neutrophil-to-Lymphocyte Ratio
DM: Diabetes Mellitus
DT: Decision Tree
eGFR: Estimated Glomerular Filtration Rate
FIB: Fibrinogen
FT3: Free Triiodothyronine
FT4: Free Thyroxine
GGT: Gamma-Glutamyl Transferase
Glu: Glucose
Hb: Hemoglobin
HbA1c: Glycated Hemoglobin
Hct: Hematocrit
HCY: Homocysteine
HDL-C: High-density Lipoprotein Cholesterol
hs-CRP: High-sensitivity C-reactive Protein
INR: International Normalized Ratio
K: Potassium
LDL-C: Low-density Lipoprotein Cholesterol
LightGBM: Light Gradient Boosting Machine
LR: Logistic Regression
LYM: Lymphocyte Count
MCH: Mean Corpuscular Hemoglobin
MCHC: Mean Corpuscular Hemoglobin Concentration
MCV: Mean Corpuscular Volume
MHR: Monocyte-to-High-density lipoprotein Cholesterol Ratio
MLR: Monocyte-to-Lymphocyte Ratio
MM: Muscle Mass
MONO: Monocyte Count
MPV: Mean Platelet Volume
Na: Sodium
NEUT: Neutrophil Count
NHHR: Non-HDL-C to HDL-C Ratio
NLR: Neutrophil-to-Lymphocyte Ratio
NMLR: Neutrophil-Monocyte-to-Lymphocyte Ratio
nonHDL-C: Non-High-Density Lipoprotein Cholesterol
Non-CP: Non-Carotid Plaque
NPV: Negative Predictive Value
NSE: Neuron-Specific Enolase
PA: Prothrombin Activity
PHOS: Phosphorus
PHR: Platelet-to-Hemoglobin Ratio
PLR: Platelet-to-Lymphocyte Ratio
PPV: Positive Predictive Value
PLT: Platelet Count
PT: Prothrombin Time
RBC: Red Blood Cell Count
RCII: Residual Cholesterol Inflammatory Index
RDW: Red Cell Distribution Width
RF: Random Forest
RFE: Recursive Feature Elimination
RLP-C: Remnant Lipoprotein Cholesterol
SBP: Systolic Blood Pressure
sd-LDL-C: Small Dense Low-Density Lipoprotein Cholesterol
SHR: Stress Hyperglycemia Ratio
SIRI: Systemic Inflammatory Response Index
SII: Systemic Immune-Inflammation Index
SVM: Support Vector Machine
SE: standard error
T3: Triiodothyronine
T4: Thyroxine
TB: Total Bilirubin
TG: Triglyceride
TgAb: Thyroglobulin Antibody
TP: Total Protein
TPOAb: Thyroid Peroxidase Antibody
TSH: Thyroid Stimulating Hormone
TyG: Triglyceride-Glucose Index
TyG-WHR: Triglyceride-Glucose-Waist-to-Hip Ratio
UACR: Urine Albumin-to-Creatinine Ratio
UA: Uric Acid
WBC: White Blood Cell Count
WC: Waist Circumference
WHR: Waist-to-Hip Ratio
XGBoost: eXtreme Gradient Boosting
β: regression coefficient
