# Supplementary material for: Development and validation of a cross-sectional risk screening nomogram for carotid plaque based on routine health examination data and the triglyceride-glucose-waist-to-hip ratio
Source: Front Endocrinol (Lausanne). 2026 Jun 17;17:1881365. doi: 10.3389/fendo.2026.1881365 (PMC13318786; doi:10.3389/fendo.2026.1881365)
Supplement: Supplementary file 1 [file DataSheet1.docx]

**Supplementary materials (merged version)**

**Supplementary Table**

| **Supplementary Table S1. Missing Ratio of Variables in the Study** | |
| --- | --- |
| Variable | Missing Ratio |
| Outcome | 0 |
| Hypertension | 0 |
| DM | 0 |
| sex | 0 |
| age | 0 |
| BMI | 0 |
| MM | 0 |
| WC | 0 |
| WHR | 0 |
| Protein | 0 |
| SBP | 0 |
| DBP | 0.000542594 |
| NLR | 0 |
| MLR | 0 |
| PLR | 0 |
| WBC | 0 |
| NEUT | 0 |
| LYM | 0 |
| MONO | 0 |
| PLT | 0 |
| RBC | 0 |
| Hb | 0 |
| MCV | 0 |
| MCHC | 0 |
| MCH | 0.000274793 |
| MPV | 0 |
| RDW | 0 |
| HCT | 0 |
| INR | 0 |
| PT | 0 |
| PA | 0 |
| APTT | 0 |
| FIB | 0 |
| PHR | 0 |
| MHR | 0 |
| TG | 0 |
| CHO | 0 |
| HDL | 0.005425935 |
| LDL | 0 |
| nonHDL | 0 |
| oxLDL-C | 0.520432692 |
| RLP | 0 |
| HbA1c | 0 |
| Glu | 0 |
| Lp(a) | 0.388020833 |
| ALT | 0 |
| AST | 0 |
| cTnI | 0.274038461 |
| cTnT | 0.338341346 |
| ALP | 0 |
| GGT | 0.001627781 |
| TP | 0 |
| ALB | 0 |
| TB | 0 |
| Mb | 0.59375 |
| DB | 0 |
| BUN | 0 |
| Cr | 0 |
| NT-proBNP | 0.113581731 |
| CK-MBmass | 0.206730769 |
| CREA | 0 |
| eGFR | 0 |
| UA | 0 |
| Ca | 0 |
| PHOS | 0 |
| K | 0.007462534 |
| Na | 0 |
| Cl | 0 |
| UACR | 0 |
| TgAb | 0 |
| TPOAb | 0.008263512 |
| T3 | 0 |
| TSH | 0 |
| FT3 | 0 |
| FT4 | 0 |
| T4 | 0 |
| HCY | 0 |
| hs_CRP | 0 |
| sdLDLC | 0 |
| TyG | 0 |
| ASBI | 0 |
| CHG | 0 |
| NHHR | 0 |
| RCII | 0 |
| CALLY | 0 |
| CLR | 0 |
| dNLR | 0 |
| NMLR | 0 |
| SIRI | 0 |
| SII | 0 |
| SHR | 0 |
| TyG_WHR | 0 |

Abbreviations: CP, Carotid Plaque; Non-CP, Non-Carotid Plaque; BMI, Body Mass Index; DM, Diabetes Mellitus; MM, Muscle Mass; BMR, Basal Metabolic Rate; WC, Waist Circumference; WHR, Waist-to-Hip Ratio; SBP, Systolic Blood Pressure; DBP, Diastolic Blood Pressure; NLR, Neutrophil-to-Lymphocyte Ratio; MLR, Monocyte-to-Lymphocyte Ratio; PLR, Platelet-to-Lymphocyte Ratio; WBC, White Blood Cell Count; NEUT, Neutrophil Count; LYM, Lymphocyte Count; MONO, Monocyte Count; PLT, Platelet Count; RBC, Red Blood Cell Count; Hb, Hemoglobin; MCV, Mean Corpuscular Volume; MCHC, Mean Corpuscular Hemoglobin Concentration; MCH, Mean Corpuscular Hemoglobin; MPV, Mean Platelet Volume; RDW, Red Cell Distribution Width; Hct, Hematocrit; INR, International Normalized Ratio; PT, Prothrombin Time; PA, Prothrombin Activity; APTT, Activated Partial Thromboplastin Time; FIB, Fibrinogen; PHR, Platelet-to-Hemoglobin Ratio; MHR, Monocyte-to-High-density lipoprotein Cholesterol Ratio; TG, Triglyceride; CHO, Total Cholesterol; HDL-C, High-density Lipoprotein Cholesterol; LDL-C, Low-density Lipoprotein Cholesterol; nonHDL-C, Non-High-Density Lipoprotein Cholesterol; RLP-C, Remnant Lipoprotein Cholesterol; HbA1c, Glycated Hemoglobin; Glu, Glucose; ALT, Alanine Aminotransferase; AST, Aspartate Aminotransferase; ALP, Alkaline Phosphatase; GGT, Gamma-Glutamyl Transferase; TP, Total Protein; ALB, Albumin; TB, Total Bilirubin; DB, Direct Bilirubin; BUN, Blood Urea Nitrogen; Cr, Creatinine; CREA, Creatinine; eGFR, Estimated Glomerular Filtration Rate; UA, Uric Acid; Ca, Calcium; PHOS, Phosphorus; K, Potassium; Na, Sodium; Cl, Chloride; UACR, Urine Albumin-to-Creatinine Ratio; TgAb, Thyroglobulin Antibody; TPOAb, Thyroid Peroxidase Antibody; T3, Triiodothyronine; TSH, Thyroid Stimulating Hormone; FT3, Free Triiodothyronine; FT4, Free Thyroxine; T4, Thyroxine; HCY, Homocysteine; hs-CRP, High-sensitivity C-reactive Protein; sd-LDL-C, Small Dense Low-Density Lipoprotein Cholesterol; TyG, Triglyceride-Glucose Index; ASBI, Atherogenic Index of Plasma; CHG, Cholesterol, high-density lipoprotein, and glucose index; NHHR, Non-HDL-C to HDL-C Ratio; RCII, Residual Cholesterol Ischemic Index; CALLY, C-Reactive Protein-Albumin-Lymphocyte Ratio; CLR, C-Reactive Protein to Lymphocyte Ratio; dNLR, Derived Neutrophil-to-Lymphocyte Ratio; NMLR, Neutrophil-Monocyte-to-Lymphocyte Ratio; SIRI, Systemic Inflammatory Response Index; SII, Systemic Immune-Inflammation Index; SHR, Stress Hyperglycemia Ratio; TyG-WHR, Triglyceride-Glucose-Waist-to-Hip Ratio.

| **Supplementary Table S2. Baseline Characteristics of the Training Set** | | | | |  |
| --- | --- | --- | --- | --- | --- |
|  |  |  |  |  |  |
| Variables | Total(n=3496) | Non-CP(n=2411) | CP(n=1085) | *p* |  |
| Hypertension | 1105 (31.61) | 668 (27.71) | 437 (40.28) | <0.001 |  |
| DM | 797 (22.80) | 458 (19.00) | 339 (31.24) | <0.001 |  |
| Sex |  |  |  | 0.004 |  |
| Male | 2399 (68.62) | 1692 (70.18) | 707 (65.16) |  |  |
| Female | 1097 (31.38) | 719 (29.82) | 378 (34.84) |  |  |
| Age | 50.00 [45.00, 56.00] | 48.00 [42.00, 53.00] | 55.00 [50.00, 61.00] | <0.001 |  |
| BMI | 25.10 [22.90, 27.30] | 24.80 [22.50, 26.90] | 25.70 [23.80, 27.90] | <0.001 |  |
| MM (Kg) | 49.00 [40.60, 54.30] | 48.30 [39.70, 53.80] | 50.10 [43.60, 55.10] | <0.001 |  |
| WC(cm) | 87.00 [81.00, 94.00] | 86.00 [79.10, 93.00] | 90.00 [84.00, 96.00] | <0.001 |  |
| WHR | 0.93 [0.85, 0.96] | 0.92 [0.83, 0.95] | 0.94 [0.90, 0.98] | <0.001 |  |
| Protein(g/dL) | 10.70 [8.80, 11.90] | 10.60 [8.60, 11.80] | 10.90 [9.40, 12.00] | <0.001 |  |
| SBP(mmHg) | 124.00 [113.00, 134.00] | 122.00 [111.00, 131.00] | 129.00 [119.00, 139.00] | <0.001 |  |
| DBP(mmHg) | 84.00 [75.00, 91.00] | 83.00 [74.00, 90.00] | 86.00 [78.00, 92.00] | <0.001 |  |
| NLR | 1.79 [1.41, 2.27] | 1.77 [1.40, 2.25] | 1.83 [1.43, 2.31] | 0.111 |  |
| MLR | 0.17 [0.14, 0.21] | 0.17 [0.14, 0.21] | 0.18 [0.14, 0.22] | 0.001 |  |
| PLR | 123.97 [99.39, 150.62] | 125.50 [100.89, 151.62] | 119.01 [95.50, 148.24] | <0.001 |  |
| WBC(×10^9/L) | 5.70 [4.91, 6.72] | 5.67 [4.87, 6.65] | 5.80 [4.99, 6.83] | 0.002 |  |
| NEUT(×10^9/L) | 3.30 [2.71, 4.06] | 3.27 [2.70, 4.00] | 3.37 [2.73, 4.20] | 0.019 |  |
| LYM(×10^9/L) | 1.84 [1.52, 2.22] | 1.84 [1.52, 2.20] | 1.83 [1.53, 2.25] | 0.402 |  |
| MONO(×10^9/L) | 0.32 [0.26, 0.39] | 0.31 [0.26, 0.38] | 0.33 [0.27, 0.40] | <0.001 |  |
| PLT(×10^9/L) | 228.00 [194.00, 264.00] | 230.00 [197.00, 265.00] | 221.00 [188.00, 260.00] | <0.001 |  |
| RBC(×10^12/L) | 4.83 [4.50, 5.12] | 4.83 [4.48, 5.13] | 4.82 [4.54, 5.11] | 0.611 |  |
| Hb(g/L) | 148.00 [137.00, 157.00] | 148.00 [135.00, 157.00] | 149.00 [140.00, 157.00] | 0.002 |  |
| MCV(fL) | 89.30 [87.00, 91.80] | 89.20 [86.90, 91.60] | 89.60 [87.40, 92.00] | <0.001 |  |
| MCHC(g/L) | 342.00 [336.00, 348.00] | 342.00 [335.00, 348.00] | 343.00 [337.00, 349.00] | <0.001 |  |
| MCH(pg) | 30.60 [29.70, 31.50] | 30.60 [29.60, 31.50] | 30.80 [29.90, 31.70] | <0.001 |  |
| MPV(fL) | 10.20 [9.70, 10.80] | 10.20 [9.70, 10.90] | 10.20 [9.60, 10.80] | 0.237 |  |
| RDW-CV(％) | 12.30 [11.90, 12.70] | 12.30 [11.90, 12.70] | 12.30 [11.90, 12.70] | 0.907 |  |
| Hct | 0.43 [0.40, 0.46] | 0.43 [0.40, 0.46] | 0.43 [0.41, 0.46] | 0.024 |  |
| INR | 0.95 [0.91, 0.99] | 0.95 [0.91, 0.99] | 0.94 [0.91, 0.99] | 0.35 |  |
| PT(Sec) | 12.70 [12.30, 13.10] | 12.70 [12.30, 13.10] | 12.60 [12.30, 13.10] | 0.409 |  |
| PA | 109.00 [102.00, 118.00] | 109.00 [102.00, 118.00] | 111.00 [102.00, 120.00] | 0.31 |  |
| APTT(Sec) | 34.60 [32.50, 36.90] | 34.70 [32.60, 37.00] | 34.40 [32.40, 36.70] | 0.028 |  |
| FIB(g/L) | 3.04 [2.73, 3.41] | 3.01 [2.70, 3.35] | 3.12 [2.79, 3.57] | <0.001 |  |
| PHR | 181.79 [144.12, 226.97] | 181.15 [142.91, 226.32] | 183.08 [146.39, 227.96] | 0.212 |  |
| MHR | 0.26 [0.19, 0.34] | 0.25 [0.18, 0.33] | 0.28 [0.20, 0.36] | <0.001 |  |
| TG(mmol/L) | 1.41 [1.00, 2.09] | 1.36 [0.95, 2.04] | 1.54 [1.11, 2.22] | <0.001 |  |
| CHO(mmol/L) | 4.63 [4.10, 5.22] | 4.60 [4.10, 5.18] | 4.72 [4.09, 5.37] | 0.02 |  |
| HDL-C(mmol/L) | 1.24 [1.04, 1.50] | 1.27 [1.05, 1.53] | 1.21 [1.02, 1.43] | <0.001 |  |
| LDL-C(mmol/L) | 2.99 [2.50, 3.53] | 2.96 [2.51, 3.46] | 3.09 [2.49, 3.65] | 0.003 |  |
| nonHDL-C(mmol/L) | 3.34 [2.81, 3.91] | 3.30 [2.80, 3.86] | 3.46 [2.86, 4.10] | <0.001 |  |
| RLP-C(mmol/L) | 0.23 [0.10, 0.45] | 0.21 [0.09, 0.43] | 0.27 [0.13, 0.48] | <0.001 |  |
| HbA1c | 5.80 [5.60, 6.10] | 5.70 [5.50, 6.00] | 5.90 [5.70, 6.30] | <0.001 |  |
| Glu(mmol/L) | 5.46 [5.11, 5.94] | 5.37 [5.07, 5.80] | 5.69 [5.26, 6.31] | <0.001 |  |
| ALT(U/L) | 19.20 [13.70, 28.02] | 18.70 [13.20, 27.80] | 20.10 [14.90, 28.60] | <0.001 |  |
| AST(U/L) | 18.30 [15.50, 22.30] | 18.00 [15.20, 21.90] | 19.10 [16.20, 22.80] | <0.001 |  |
| ALP(U/L) | 62.25 [52.70, 74.10] | 61.40 [52.00, 72.60] | 64.80 [54.80, 77.00] | <0.001 |  |
| GGT(U/L) | 25.90 [16.50, 43.90] | 24.90 [15.70, 43.20] | 28.00 [18.90, 46.60] | <0.001 |  |
| TP(g/L) | 72.90 [70.30, 75.60] | 73.00 [70.30, 75.70] | 72.70 [70.30, 75.10] | 0.046 |  |
| ALB(g/L) | 46.40 [44.70, 48.00] | 46.40 [44.80, 48.10] | 46.30 [44.70, 47.90] | 0.173 |  |
| TB(umol/L) | 11.50 [8.70, 15.30] | 11.30 [8.45, 15.10] | 12.00 [9.20, 15.50] | <0.001 |  |
| DB(umol/L) | 3.90 [3.10, 5.00] | 3.90 [3.00, 4.90] | 4.00 [3.20, 5.10] | <0.001 |  |
| BUN(mmol/L) | 4.94 [4.24, 5.77] | 4.89 [4.16, 5.70] | 5.10 [4.41, 5.93] | <0.001 |  |
| Cr(mg/dL) | 0.81 [0.69, 0.92] | 0.80 [0.68, 0.92] | 0.83 [0.72, 0.93] | <0.001 |  |
| CREA(umol/L) | 71.50 [61.00, 81.40] | 70.50 [60.00, 81.10] | 73.30 [64.00, 82.20] | <0.001 |  |
| eGFR(mL/min/1.73m^2) | 101.37 [93.13, 107.75] | 103.06 [94.71, 109.57] | 97.80 [90.04, 103.78] | <0.001 |  |
| UA(umol/L) | 343.05 [282.03, 403.70] | 337.90 [276.90, 400.50] | 355.20 [296.60, 410.70] | <0.001 |  |
| Ca(mmol/L) | 2.34 [2.28, 2.39] | 2.34 [2.28, 2.39] | 2.34 [2.29, 2.39] | 0.261 |  |
| PHOS(mmol/L) | 1.16 [1.07, 1.26] | 1.16 [1.07, 1.26] | 1.16 [1.07, 1.27] | 0.742 |  |
| K(mmol/L) | 4.32 [4.13, 4.51] | 4.31 [4.12, 4.50] | 4.35 [4.15, 4.54] | 0.003 |  |
| Na(mmol/L) | 142.00 [141.00, 144.00] | 142.00 [140.00, 144.00] | 143.00 [141.00, 144.00] | <0.001 |  |
| Cl(mmol/L) | 101.50 [99.90, 103.10] | 101.60 [99.80, 103.20] | 101.50 [100.00, 103.10] | 0.471 |  |
| UACR(mg/g) | 5.60 [4.00, 9.30] | 5.30 [3.90, 8.60] | 6.40 [4.30, 12.20] | <0.001 |  |
| TgAb(IU/mL) | 12.00 [10.20, 15.10] | 12.10 [10.20, 15.10] | 11.90 [10.10, 15.00] | 0.117 |  |
| TPOAb(IU/mL) | 13.40 [9.90, 17.70] | 13.30 [9.90, 17.85] | 13.50 [9.90, 17.50] | 0.884 |  |
| T3(nmol/L) | 1.77 [1.58, 1.97] | 1.76 [1.58, 1.96] | 1.78 [1.59, 1.98] | 0.158 |  |
| TSH(mIU/l) | 2.07 [1.44, 2.99] | 2.09 [1.46, 2.99] | 1.99 [1.41, 3.00] | 0.119 |  |
| FT3(pmol/L) | 5.08 [4.67, 5.53] | 5.08 [4.67, 5.54] | 5.07 [4.69, 5.51] | 0.768 |  |
| FT4(pmol/L) | 16.82 [15.43, 18.41] | 16.92 [15.41, 18.47] | 16.67 [15.47, 18.23] | 0.062 |  |
| T4(nmol/L) | 97.06 [86.44, 108.30] | 96.71 [86.24, 108.00] | 97.79 [86.78, 109.00] | 0.133 |  |
| HCY(umol/L) | 10.60 [8.70, 13.00] | 10.20 [8.50, 12.65] | 11.30 [9.40, 13.50] | <0.001 |  |
| hs-CRP(mg/L) | 0.08 [0.05, 0.15] | 0.08 [0.04, 0.14] | 0.09 [0.05, 0.17] | <0.001 |  |
| sd-LDL-C(mmol/L) | 9.68 [9.45, 9.92] | 9.68 [9.46, 9.90] | 9.70 [9.45, 9.97] | 0.129 |  |
| TyG | 8.75 [8.36, 9.20] | 8.69 [8.29, 9.15] | 8.89 [8.51, 9.31] | <0.001 |  |
| ASBI | 0.06 [0.06, 0.07] | 0.06 [0.06, 0.07] | 0.06 [0.06, 0.07] | 0.841 |  |
| CHG | 5.22 [4.99, 5.46] | 5.18 [4.95, 5.42] | 5.30 [5.08, 5.53] | <0.001 |  |
| NHHR | 2.69 [2.03, 3.44] | 2.63 [1.97, 3.34] | 2.85 [2.15, 3.60] | <0.001 |  |
| RCII | 0.07 [0.02, 0.21] | 0.07 [0.02, 0.20] | 0.09 [0.03, 0.26] | <0.001 |  |
| CALLY | 106.30 [56.16, 183.77] | 110.66 [59.53, 191.89] | 93.10 [49.36, 159.89] | <0.001 |  |
| CLR | 0.04 [0.03, 0.08] | 0.04 [0.02, 0.08] | 0.05 [0.03, 0.09] | <0.001 |  |
| dNLR | 0.87 [0.85, 0.90] | 0.88 [0.85, 0.90] | 0.87 [0.84, 0.89] | 0.004 |  |
| NMLR | 1.97 [1.56, 2.48] | 1.94 [1.56, 2.45] | 2.02 [1.58, 2.51] | 0.077 |  |
| SIRI | 0.57 [0.41, 0.80] | 0.56 [0.40, 0.77] | 0.59 [0.42, 0.85] | 0.001 |  |
| SII | 406.08 [305.53, 535.93] | 406.55 [308.73, 536.58] | 405.89 [297.65, 533.33] | 0.321 |  |
| SHR | 0.83 [0.78, 0.89] | 0.83 [0.78, 0.89] | 0.83 [0.78, 0.89] | 0.561 |  |
| TyG_WHR | 8.06 [7.28, 8.72] | 7.93 [7.06, 8.59] | 8.37 [7.74, 9.01] | <0.001 |  |

Abbreviations: CP, Carotid Plaque; Non-CP, Non-Carotid Plaque; BMI, Body Mass Index; DM, Diabetes Mellitus; MM, Muscle Mass; BMR, Basal Metabolic Rate; WC, Waist Circumference; WHR, Waist-to-Hip Ratio; SBP, Systolic Blood Pressure; DBP, Diastolic Blood Pressure; NLR, Neutrophil-to-Lymphocyte Ratio; MLR, Monocyte-to-Lymphocyte Ratio; PLR, Platelet-to-Lymphocyte Ratio; WBC, White Blood Cell Count; NEUT, Neutrophil Count; LYM, Lymphocyte Count; MONO, Monocyte Count; PLT, Platelet Count; RBC, Red Blood Cell Count; Hb, Hemoglobin; MCV, Mean Corpuscular Volume; MCHC, Mean Corpuscular Hemoglobin Concentration; MCH, Mean Corpuscular Hemoglobin; MPV, Mean Platelet Volume; RDW, Red Cell Distribution Width; Hct, Hematocrit; INR, International Normalized Ratio; PT, Prothrombin Time; PA, Prothrombin Activity; APTT, Activated Partial Thromboplastin Time; FIB, Fibrinogen; PHR, Platelet-to-Hemoglobin Ratio; MHR, Monocyte-to-High-density lipoprotein Cholesterol Ratio; TG, Triglyceride; CHO, Total Cholesterol; HDL-C, High-density Lipoprotein Cholesterol; LDL-C, Low-density Lipoprotein Cholesterol; nonHDL-C, Non-High-Density Lipoprotein Cholesterol; RLP-C, Remnant Lipoprotein Cholesterol; HbA1c, Glycated Hemoglobin; Glu, Glucose; ALT, Alanine Aminotransferase; AST, Aspartate Aminotransferase; ALP, Alkaline Phosphatase; GGT, Gamma-Glutamyl Transferase; TP, Total Protein; ALB, Albumin; TB, Total Bilirubin; DB, Direct Bilirubin; BUN, Blood Urea Nitrogen; Cr, Creatinine; CREA, Creatinine; eGFR, Estimated Glomerular Filtration Rate; UA, Uric Acid; Ca, Calcium; PHOS, Phosphorus; K, Potassium; Na, Sodium; Cl, Chloride; UACR, Urine Albumin-to-Creatinine Ratio; TgAb, Thyroglobulin Antibody; TPOAb, Thyroid Peroxidase Antibody; T3, Triiodothyronine; TSH, Thyroid Stimulating Hormone; FT3, Free Triiodothyronine; FT4, Free Thyroxine; T4, Thyroxine; HCY, Homocysteine; hs-CRP, High-sensitivity C-reactive Protein; sd-LDL-C, Small Dense Low-Density Lipoprotein Cholesterol; TyG, Triglyceride-Glucose Index; ASBI, Atherogenic Index of Plasma; CHG, Cholesterol, high-density lipoprotein, and glucose index; NHHR, Non-HDL-C to HDL-C Ratio; RCII, Residual Cholesterol Ischemic Index; CALLY, C-Reactive Protein-Albumin-Lymphocyte Ratio; CLR, C-Reactive Protein to Lymphocyte Ratio; dNLR, Derived Neutrophil-to-Lymphocyte Ratio; NMLR, Neutrophil-Monocyte-to-Lymphocyte Ratio; SIRI, Systemic Inflammatory Response Index; SII, Systemic Immune-Inflammation Index; SHR, Stress Hyperglycemia Ratio; TyG-WHR, Triglyceride-Glucose-Waist-to-Hip Ratio.

| **Supplementary Table S3. Baseline Characteristics of the** **Validation Set** | | | | |  |
| --- | --- | --- | --- | --- | --- |
|  |  |  |  |  |  |
| Variables | Total(n=1496) | Non-CP(n=1032) | CP(n=464) | *p* |  |
| Hypertension | 502 (33.56) | 305 (29.55) | 197 (42.46) | <0.001 |  |
| DM | 352 (23.53) | 198 (19.19) | 154 (33.19) | <0.001 |  |
| Sex |  |  |  | 0.687 |  |
| Male | 1028 (68.72) | 713 (69.09) | 315 (67.89) |  |  |
| Female | 468 (31.28) | 319 (30.91) | 149 (32.11) |  |  |
| Age | 50.00 [44.00, 56.00] | 48.00 [42.00, 53.00] | 55.50 [51.00, 60.00] | <0.001 |  |
| BMI | 24.90 [22.80, 27.10] | 24.60 [22.40, 26.80] | 25.60 [23.70, 27.50] | <0.001 |  |
| MM (Kg) | 48.70 [40.10, 54.32] | 48.40 [39.50, 54.30] | 49.10 [42.20, 54.40] | 0.02 |  |
| WC(cm) | 87.00 [80.00, 94.00] | 86.00 [79.00, 93.00] | 90.00 [84.00, 95.20] | <0.001 |  |
| WHR | 0.92 [0.84, 0.96] | 0.91 [0.82, 0.95] | 0.94 [0.89, 0.98] | <0.001 |  |
| Protein(g/dL) | 10.70 [8.60, 11.90] | 10.55 [8.50, 11.90] | 10.80 [9.00, 11.90] | 0.058 |  |
| SBP(mmHg) | 124.00 [115.00, 135.00] | 122.00 [113.00, 132.00] | 130.50 [120.00, 138.25] | <0.001 |  |
| DBP(mmHg) | 84.00 [76.00, 91.00] | 83.00 [74.00, 90.00] | 87.00 [79.00, 93.00] | <0.001 |  |
| NLR | 1.78 [1.42, 2.24] | 1.76 [1.42, 2.22] | 1.81 [1.43, 2.32] | 0.157 |  |
| MLR | 0.17 [0.14, 0.21] | 0.17 [0.14, 0.21] | 0.17 [0.14, 0.21] | 0.017 |  |
| PLR | 123.24 [101.59, 151.54] | 125.71 [103.77, 153.32] | 119.43 [94.51, 147.27] | 0.001 |  |
| WBC(×10^9/L) | 5.69 [4.87, 6.76] | 5.62 [4.80, 6.61] | 5.90 [5.02, 7.08] | <0.001 |  |
| NEUT(×10^9/L) | 3.32 [2.65, 4.09] | 3.25 [2.62, 4.00] | 3.45 [2.74, 4.29] | 0.004 |  |
| LYM(×10^9/L) | 1.85 [1.53, 2.21] | 1.84 [1.52, 2.20] | 1.89 [1.58, 2.27] | 0.092 |  |
| MONO(×10^9/L) | 0.32 [0.25, 0.39] | 0.31 [0.25, 0.38] | 0.33 [0.27, 0.40] | <0.001 |  |
| PLT(×10^9/L) | 227.00 [199.00, 262.00] | 229.50 [201.00, 265.25] | 219.50 [192.00, 253.25] | 0.001 |  |
| RBC(×10^12/L) | 4.81 [4.49, 5.12] | 4.80 [4.46, 5.11] | 4.84 [4.54, 5.14] | 0.022 |  |
| Hb(g/L) | 147.00 [136.00, 156.25] | 147.00 [135.00, 156.00] | 149.00 [139.00, 157.00] | 0.002 |  |
| MCV(fL) | 89.10 [86.90, 91.43] | 89.00 [86.80, 91.30] | 89.50 [87.30, 92.00] | 0.004 |  |
| MCHC(g/L) | 342.00 [335.00, 348.00] | 342.00 [335.00, 348.00] | 342.00 [335.00, 348.00] | 0.9 |  |
| MCH(pg) | 30.50 [29.60, 31.40] | 30.50 [29.60, 31.40] | 30.50 [29.60, 31.60] | 0.181 |  |
| MPV(fL) | 10.20 [9.70, 10.90] | 10.30 [9.70, 10.90] | 10.20 [9.60, 10.90] | 0.23 |  |
| RDW-CV(％) | 12.30 [11.90, 12.70] | 12.30 [11.90, 12.70] | 12.30 [12.00, 12.70] | 0.191 |  |
| Hct | 0.43 [0.40, 0.46] | 0.43 [0.40, 0.45] | 0.44 [0.41, 0.46] | <0.001 |  |
| INR | 0.95 [0.91, 0.99] | 0.95 [0.91, 0.99] | 0.94 [0.91, 0.99] | 0.327 |  |
| PT(Sec) | 12.70 [12.30, 13.10] | 12.70 [12.30, 13.10] | 12.70 [12.20, 13.10] | 0.3 |  |
| PA | 109.00 [102.00, 118.00] | 109.00 [102.00, 118.00] | 111.00 [102.00, 118.00] | 0.35 |  |
| APTT(Sec) | 34.60 [32.70, 37.10] | 34.60 [32.70, 37.20] | 34.60 [32.60, 36.90] | 0.327 |  |
| FIB(g/L) | 3.05 [2.72, 3.43] | 3.01 [2.67, 3.34] | 3.16 [2.84, 3.62] | <0.001 |  |
| PHR | 181.25 [142.53, 228.08] | 183.35 [143.18, 230.24] | 176.51 [140.99, 223.28] | 0.239 |  |
| MHR | 0.25 [0.19, 0.34] | 0.25 [0.18, 0.33] | 0.27 [0.20, 0.36] | <0.001 |  |
| TG(mmol/L) | 1.42 [1.00, 2.13] | 1.36 [0.97, 2.13] | 1.54 [1.11, 2.13] | 0.003 |  |
| CHO(mmol/L) | 4.69 [4.08, 5.30] | 4.68 [4.10, 5.26] | 4.78 [4.02, 5.37] | 0.331 |  |
| HDL-C(mmol/L) | 1.25 [1.05, 1.50] | 1.25 [1.06, 1.51] | 1.23 [1.04, 1.45] | 0.108 |  |
| LDL-C(mmol/L) | 3.01 [2.46, 3.56] | 3.00 [2.48, 3.50] | 3.05 [2.40, 3.67] | 0.174 |  |
| nonHDL-C(mmol/L) | 3.40 [2.80, 3.96] | 3.38 [2.81, 3.93] | 3.49 [2.78, 4.12] | 0.139 |  |
| RLP-C(mmol/L) | 0.23 [0.10, 0.48] | 0.22 [0.09, 0.46] | 0.25 [0.13, 0.49] | 0.054 |  |
| HbA1c | 5.80 [5.60, 6.10] | 5.70 [5.50, 6.00] | 6.00 [5.70, 6.40] | <0.001 |  |
| Glu(mmol/L) | 5.49 [5.13, 5.93] | 5.39 [5.06, 5.81] | 5.71 [5.29, 6.38] | <0.001 |  |
| ALT(U/L) | 19.00 [14.00, 27.70] | 18.50 [13.60, 27.33] | 19.90 [14.97, 28.52] | 0.007 |  |
| AST(U/L) | 18.40 [15.40, 22.00] | 18.10 [15.30, 21.70] | 19.00 [15.80, 22.63] | 0.006 |  |
| ALP(U/L) | 62.50 [52.90, 76.00] | 61.50 [52.27, 74.62] | 65.35 [55.70, 80.82] | <0.001 |  |
| GGT(U/L) | 26.80 [16.70, 44.55] | 25.75 [15.57, 44.55] | 27.70 [19.00, 44.60] | 0.01 |  |
| TP(g/L) | 73.20 [70.50, 75.70] | 73.10 [70.60, 75.70] | 73.20 [70.10, 75.70] | 0.452 |  |
| ALB(g/L) | 46.60 [44.80, 48.10] | 46.70 [44.90, 48.20] | 46.20 [44.80, 47.90] | 0.036 |  |
| TB(umol/L) | 11.50 [8.70, 14.90] | 11.40 [8.60, 14.70] | 11.90 [8.90, 15.20] | 0.142 |  |
| DB(umol/L) | 3.90 [3.10, 4.90] | 3.80 [3.10, 4.80] | 4.00 [3.10, 5.10] | 0.025 |  |
| BUN(mmol/L) | 4.97 [4.21, 5.82] | 4.92 [4.16, 5.73] | 5.11 [4.35, 6.06] | <0.001 |  |
| Cr(mg/dL) | 0.80 [0.68, 0.92] | 0.79 [0.67, 0.92] | 0.82 [0.70, 0.93] | 0.017 |  |
| CREA(umol/L) | 70.45 [60.27, 81.30] | 69.65 [59.10, 80.90] | 72.15 [61.90, 82.03] | 0.017 |  |
| eGFR(mL/min/1.73m^2) | 101.38 [92.99, 108.22] | 102.72 [94.56, 109.80] | 98.46 [90.21, 104.85] | <0.001 |  |
| UA(umol/L) | 340.45 [279.65, 406.02] | 333.85 [271.58, 402.20] | 350.40 [297.70, 411.70] | 0.001 |  |
| Ca(mmol/L) | 2.35 [2.29, 2.40] | 2.35 [2.29, 2.40] | 2.35 [2.29, 2.40] | 0.222 |  |
| PHOS(mmol/L) | 1.17 [1.07, 1.27] | 1.17 [1.07, 1.27] | 1.17 [1.06, 1.26] | 0.78 |  |
| K(mmol/L) | 4.33 [4.16, 4.53] | 4.33 [4.15, 4.53] | 4.34 [4.16, 4.52] | 0.658 |  |
| Na(mmol/L) | 142.00 [141.00, 144.00] | 142.00 [140.75, 144.00] | 143.00 [141.00, 144.00] | 0.01 |  |
| Cl(mmol/L) | 101.50 [99.90, 103.20] | 101.60 [99.97, 103.20] | 101.25 [99.70, 103.20] | 0.248 |  |
| UACR(mg/g) | 5.60 [4.10, 9.10] | 5.30 [4.00, 8.70] | 6.35 [4.30, 10.62] | <0.001 |  |
| TgAb(IU/mL) | 12.10 [10.10, 15.12] | 12.20 [10.10, 15.40] | 11.80 [10.00, 14.70] | 0.107 |  |
| TPOAb(IU/mL) | 13.50 [9.90, 17.80] | 13.40 [9.90, 17.60] | 13.95 [9.90, 18.60] | 0.306 |  |
| T3(nmol/L) | 1.76 [1.59, 1.96] | 1.75 [1.58, 1.95] | 1.78 [1.61, 1.99] | 0.03 |  |
| TSH(mIU/l) | 2.11 [1.49, 3.00] | 2.14 [1.54, 3.01] | 2.02 [1.37, 2.99] | 0.023 |  |
| FT3(pmol/L) | 5.09 [4.66, 5.53] | 5.08 [4.63, 5.54] | 5.10 [4.75, 5.49] | 0.497 |  |
| FT4(pmol/L) | 16.83 [15.44, 18.45] | 16.84 [15.44, 18.39] | 16.78 [15.43, 18.61] | 0.941 |  |
| T4(nmol/L) | 97.32 [86.26, 108.40] | 97.34 [86.26, 107.50] | 97.19 [86.30, 109.82] | 0.286 |  |
| HCY(umol/L) | 10.40 [8.70, 12.72] | 10.20 [8.40, 12.50] | 10.70 [9.20, 13.10] | 0.001 |  |
| hs-CRP(mg/L) | 0.08 [0.05, 0.15] | 0.08 [0.04, 0.15] | 0.10 [0.05, 0.18] | <0.001 |  |
| sd-LDL-C(mmol/L) | 9.69 (0.38) | 9.68 (0.38) | 9.69 (0.39) | 0.813 |  |
| TyG | 8.76 [8.37, 9.21] | 8.70 [8.29, 9.18] | 8.88 [8.50, 9.26] | <0.001 |  |
| ASBI | 0.06 [0.06, 0.07] | 0.06 [0.06, 0.07] | 0.06 [0.06, 0.06] | 0.867 |  |
| CHG | 5.24 [4.98, 5.47] | 5.20 [4.95, 5.44] | 5.29 [5.07, 5.54] | <0.001 |  |
| NHHR | 2.70 [2.01, 3.48] | 2.69 [1.97, 3.45] | 2.73 [2.11, 3.53] | 0.095 |  |
| RCII | 0.08 [0.02, 0.23] | 0.07 [0.02, 0.22] | 0.10 [0.04, 0.25] | <0.001 |  |
| CALLY | 103.21 [56.32, 181.30] | 111.70 [59.67, 194.94] | 91.17 [50.68, 159.07] | <0.001 |  |
| CLR | 0.04 [0.03, 0.08] | 0.04 [0.02, 0.08] | 0.05 [0.03, 0.09] | <0.001 |  |
| dNLR | 0.88 [0.85, 0.90] | 0.88 [0.85, 0.90] | 0.88 [0.85, 0.90] | 0.223 |  |
| NMLR | 1.94 [1.57, 2.45] | 1.93 [1.57, 2.42] | 1.99 [1.58, 2.52] | 0.131 |  |
| SIRI | 0.56 [0.41, 0.80] | 0.54 [0.40, 0.77] | 0.60 [0.41, 0.86] | 0.002 |  |
| SII | 406.76 [304.50, 534.07] | 405.91 [307.70, 528.69] | 411.85 [299.33, 551.84] | 0.716 |  |
| SHR | 0.83 [0.78, 0.89] | 0.83 [0.78, 0.88] | 0.84 [0.78, 0.90] | 0.202 |  |
| TyG-WHR | 8.05 [7.20, 8.74] | 7.84 [7.02, 8.63] | 8.30 [7.75, 8.92] | <0.001 |  |

Abbreviations: CP, Carotid Plaque; Non-CP, Non-Carotid Plaque; BMI, Body Mass Index; DM, Diabetes Mellitus; MM, Muscle Mass; BMR, Basal Metabolic Rate; WC, Waist Circumference; WHR, Waist-to-Hip Ratio; SBP, Systolic Blood Pressure; DBP, Diastolic Blood Pressure; NLR, Neutrophil-to-Lymphocyte Ratio; MLR, Monocyte-to-Lymphocyte Ratio; PLR, Platelet-to-Lymphocyte Ratio; WBC, White Blood Cell Count; NEUT, Neutrophil Count; LYM, Lymphocyte Count; MONO, Monocyte Count; PLT, Platelet Count; RBC, Red Blood Cell Count; Hb, Hemoglobin; MCV, Mean Corpuscular Volume; MCHC, Mean Corpuscular Hemoglobin Concentration; MCH, Mean Corpuscular Hemoglobin; MPV, Mean Platelet Volume; RDW, Red Cell Distribution Width; Hct, Hematocrit; INR, International Normalized Ratio; PT, Prothrombin Time; PA, Prothrombin Activity; APTT, Activated Partial Thromboplastin Time; FIB, Fibrinogen; PHR, Platelet-to-Hemoglobin Ratio; MHR, Monocyte-to-High-density lipoprotein Cholesterol Ratio; TG, Triglyceride; CHO, Total Cholesterol; HDL-C, High-density Lipoprotein Cholesterol; LDL-C, Low-density Lipoprotein Cholesterol; nonHDL-C, Non-High-Density Lipoprotein Cholesterol; RLP-C, Remnant Lipoprotein Cholesterol; HbA1c, Glycated Hemoglobin; Glu, Glucose; ALT, Alanine Aminotransferase; AST, Aspartate Aminotransferase; ALP, Alkaline Phosphatase; GGT, Gamma-Glutamyl Transferase; TP, Total Protein; ALB, Albumin; TB, Total Bilirubin; DB, Direct Bilirubin; BUN, Blood Urea Nitrogen; Cr, Creatinine; CREA, Creatinine; eGFR, Estimated Glomerular Filtration Rate; UA, Uric Acid; Ca, Calcium; PHOS, Phosphorus; K, Potassium; Na, Sodium; Cl, Chloride; UACR, Urine Albumin-to-Creatinine Ratio; TgAb, Thyroglobulin Antibody; TPOAb, Thyroid Peroxidase Antibody; T3, Triiodothyronine; TSH, Thyroid Stimulating Hormone; FT3, Free Triiodothyronine; FT4, Free Thyroxine; T4, Thyroxine; HCY, Homocysteine; hs-CRP, High-sensitivity C-reactive Protein; sd-LDL-C, Small Dense Low-Density Lipoprotein Cholesterol; TyG, Triglyceride-Glucose Index; ASBI, Atherogenic Index of Plasma; CHG, Cholesterol, high-density lipoprotein, and glucose index; NHHR, Non-HDL-C to HDL-C Ratio; RCII, Residual Cholesterol Ischemic Index; CALLY, C-Reactive Protein-Albumin-Lymphocyte Ratio; CLR, C-Reactive Protein to Lymphocyte Ratio; dNLR, Derived Neutrophil-to-Lymphocyte Ratio; NMLR, Neutrophil-Monocyte-to-Lymphocyte Ratio; SIRI, Systemic Inflammatory Response Index; SII, Systemic Immune-Inflammation Index; SHR, Stress Hyperglycemia Ratio; TyG-WHR, Triglyceride-Glucose-Waist-to-Hip Ratio; .

| **Supplementary Table S4. Baseline Characteristics of the Temporal validation set** | | | | |  |
| --- | --- | --- | --- | --- | --- |
|  |  |  |  |  |  |
| Variables | Total(n=3812) | Non-CP(n=1870) | CP(n=1942) | *p* |  |
| Hypertension | 1052 (27.60) | 178 ( 9.52) | 874 (45.01) | <0.001 |  |
| DM | 689 (18.07) | 167 ( 8.93) | 522 (26.88) | <0.001 |  |
| Sex |  |  |  | <0.001 |  |
| Male | 2110 (55.35) | 1093 (58.45) | 1017 (52.37) |  |  |
| Female | 1702 (44.65) | 777 (41.55) | 925 (47.63) |  |  |
| Age | 51.00 [44.00, 61.00] | 45.00 [41.00, 52.00] | 58.00 [50.00, 68.00] | <0.001 |  |
| BMI | 24.80 [22.70, 27.13] | 23.90 [21.80, 26.10] | 25.65 [23.60, 28.00] | <0.001 |  |
| MM (Kg) | 48.10 [40.10, 54.30] | 47.05 [39.50, 53.30] | 49.00 [41.20, 55.10] | <0.001 |  |
| WC(cm) | 89.00 [81.00, 95.00] | 85.00 [78.00, 92.00] | 91.00 [85.00, 97.00] | <0.001 |  |
| WHR | 0.91 [0.83, 0.95] | 0.87 [0.80, 0.93] | 0.93 [0.86, 0.97] | <0.001 |  |
| Protein(g/dL) | 10.60 [8.70, 12.00] | 10.40 [8.60, 11.80] | 10.80 [8.90, 12.10] | <0.001 |  |
| SBP(mmHg) | 116.00 [115.00, 137.00] | 115.00 [107.00, 116.00] | 134.00 [116.00, 164.00] | <0.001 |  |
| DBP(mmHg) | 80.00 [74.00, 89.00] | 77.00 [71.00, 82.00] | 85.00 [78.00, 96.00] | <0.001 |  |
| NLR | 1.81 [1.43, 2.32] | 1.78 [1.41, 2.24] | 1.85 [1.46, 2.40] | <0.001 |  |
| MLR | 0.17 [0.13, 0.21] | 0.16 [0.13, 0.20] | 0.17 [0.14, 0.21] | <0.001 |  |
| PLR | 116.31 [94.14, 144.59] | 120.75 [99.14, 148.68] | 111.86 [89.72, 139.72] | <0.001 |  |
| WBC(×10^9/L) | 5.83 [4.92, 6.91] | 5.61 [4.71, 6.63] | 6.06 [5.12, 7.12] | <0.001 |  |
| NEUT(×10^9/L) | 3.40 [2.75, 4.26] | 3.25 [2.61, 4.06] | 3.54 [2.88, 4.41] | <0.001 |  |
| LYM(×10^9/L) | 1.87 [1.53, 2.24] | 1.83 [1.52, 2.17] | 1.91 [1.55, 2.32] | <0.001 |  |
| MONO(×10^9/L) | 0.31 [0.25, 0.39] | 0.29 [0.24, 0.36] | 0.33 [0.26, 0.41] | <0.001 |  |
| PLT(×10^9/L) | 217.00 [186.00, 255.00] | 220.50 [189.00, 257.00] | 214.00 [182.00, 251.00] | <0.001 |  |
| RBC(×10^12/L) | 4.82 [4.50, 5.14] | 4.79 [4.45, 5.14] | 4.85 [4.55, 5.15] | 0.001 |  |
| Hb(g/L) | 149.00 [137.00, 158.00] | 147.00 [135.00, 158.00] | 150.00 [140.00, 159.00] | <0.001 |  |
| MCV(fL) | 89.20 [86.70, 91.80] | 89.10 [86.40, 91.60] | 89.40 [86.90, 91.90] | 0.001 |  |
| MCHC(g/L) | 343.00 [337.00, 350.00] | 343.00 [336.00, 349.00] | 344.00 [338.00, 351.00] | <0.001 |  |
| MCH(pg) | 30.70 [29.70, 31.60] | 30.60 [29.60, 31.50] | 30.80 [29.90, 31.80] | <0.001 |  |
| MPV(fL) | 10.40 [9.80, 11.00] | 10.40 [9.83, 11.00] | 10.40 [9.80, 11.00] | 0.501 |  |
| RDW-CV(％) | 12.60 [12.20, 13.10] | 12.60 [12.20, 13.10] | 12.70 [12.30, 13.10] | <0.001 |  |
| Hct | 0.43 [0.40, 0.46] | 0.43 [0.40, 0.46] | 0.44 [0.41, 0.46] | <0.001 |  |
| INR | 0.99 [0.95, 1.03] | 0.99 [0.95, 1.03] | 0.99 [0.94, 1.03] | 0.686 |  |
| PT(Sec) | 13.00 [12.60, 13.50] | 13.00 [12.60, 13.50] | 13.00 [12.60, 13.50] | 0.61 |  |
| PA | 102.00 [95.00, 110.00] | 102.00 [95.00, 110.00] | 102.00 [95.00, 110.00] | 0.728 |  |
| APTT(Sec) | 35.20 [33.00, 37.42] | 35.40 [33.23, 37.60] | 35.00 [32.80, 37.30] | 0.002 |  |
| FIB(g/L) | 3.11 [2.78, 3.51] | 2.99 [2.68, 3.35] | 3.24 [2.89, 3.64] | <0.001 |  |
| PHR | 173.26 [136.73, 218.94] | 169.88 [134.95, 214.37] | 175.58 [139.10, 222.45] | 0.007 |  |
| MHR | 0.25 [0.18, 0.34] | 0.23 [0.16, 0.31] | 0.27 [0.20, 0.36] | <0.001 |  |
| TG(mmol/L) | 1.36 [0.94, 1.97] | 1.22 [0.84, 1.80] | 1.48 [1.08, 2.13] | <0.001 |  |
| CHO(mmol/L) | 4.70 [4.10, 5.36] | 4.61 [4.05, 5.21] | 4.81 [4.16, 5.51] | <0.001 |  |
| HDL-C(mmol/L) | 1.24 [1.04, 1.50] | 1.29 [1.07, 1.55] | 1.21 [1.02, 1.45] | <0.001 |  |
| LDL-C(mmol/L) | 2.98 [2.42, 3.56] | 2.88 [2.40, 3.44] | 3.09 [2.49, 3.69] | <0.001 |  |
| nonHDL-C(mmol/L) | 3.40 [2.79, 4.06] | 3.26 [2.71, 3.88] | 3.56 [2.91, 4.22] | <0.001 |  |
| RLP-C(mmol/L) | 0.32 [0.18, 0.54] | 0.28 [0.15, 0.49] | 0.36 [0.20, 0.58] | <0.001 |  |
| HbA1c | 5.80 [5.50, 6.20] | 5.60 [5.40, 5.90] | 5.90 [5.60, 6.40] | <0.001 |  |
| Glu(mmol/L) | 5.47 [5.12, 6.05] | 5.28 [5.00, 5.67] | 5.70 [5.28, 6.45] | <0.001 |  |
| ALT(U/L) | 18.60 [13.70, 27.10] | 17.20 [12.62, 24.98] | 19.80 [14.40, 28.48] | <0.001 |  |
| AST(U/L) | 18.20 [15.50, 22.50] | 17.60 [14.90, 21.30] | 19.00 [16.10, 23.10] | <0.001 |  |
| ALP(U/L) | 66.10 [55.20, 79.30] | 62.60 [52.10, 75.68] | 69.45 [58.90, 82.18] | <0.001 |  |
| GGT(U/L) | 25.10 [16.30, 41.70] | 21.85 [14.30, 36.80] | 28.00 [18.90, 46.20] | <0.001 |  |
| TP(g/L) | 71.80 [69.20, 74.60] | 71.70 [69.00, 74.30] | 71.90 [69.40, 74.90] | 0.005 |  |
| ALB(g/L) | 45.90 [44.20, 47.60] | 46.00 [44.30, 47.70] | 45.70 [44.10, 47.40] | 0.001 |  |
| TB(umol/L) | 10.90 [8.30, 14.10] | 10.70 [8.03, 13.80] | 11.10 [8.43, 14.40] | 0.001 |  |
| DB(umol/L) | 3.90 [3.10, 4.90] | 3.80 [3.00, 4.80] | 4.00 [3.20, 4.97] | 0.001 |  |
| BUN(mmol/L) | 4.95 [4.22, 5.90] | 4.75 [4.05, 5.59] | 5.20 [4.43, 6.14] | <0.001 |  |
| Cr(mg/dL) | 0.79 [0.66, 0.90] | 0.77 [0.65, 0.89] | 0.81 [0.68, 0.91] | <0.001 |  |
| CREA(umol/L) | 69.80 [58.60, 79.90] | 68.20 [57.40, 78.80] | 71.30 [60.30, 80.50] | <0.001 |  |
| eGFR(mL/min/1.73m^2) | 100.69 [90.73, 108.65] | 105.03 [96.94, 112.06] | 95.33 [87.06, 104.20] | <0.001 |  |
| UA(umol/L) | 328.10 [268.80, 392.22] | 316.85 [255.33, 385.37] | 337.75 [281.60, 399.48] | <0.001 |  |
| Ca(mmol/L) | 2.34 [2.28, 2.39] | 2.33 [2.28, 2.39] | 2.34 [2.28, 2.40] | 0.012 |  |
| PHOS(mmol/L) | 1.17 [1.07, 1.27] | 1.17 [1.07, 1.27] | 1.17 [1.07, 1.27] | 0.926 |  |
| K(mmol/L) | 4.25 [4.05, 4.45] | 4.25 [4.05, 4.44] | 4.25 [4.05, 4.46] | 0.713 |  |
| Na(mmol/L) | 141.00 [140.00, 143.00] | 141.00 [140.00, 143.00] | 142.00 [140.00, 143.00] | <0.001 |  |
| Cl(mmol/L) | 102.00 [100.30, 103.60] | 102.00 [100.40, 103.60] | 101.90 [100.10, 103.60] | 0.129 |  |
| UACR(mg/g) | 6.40 [4.40, 12.03] | 5.30 [4.00, 8.20] | 8.30 [5.10, 18.90] | <0.001 |  |
| TgAb(IU/mL) | 14.70 [10.70, 21.10] | 14.60 [10.70, 20.80] | 14.70 [10.80, 21.37] | 0.531 |  |
| TPOAb(IU/mL) | 10.80 [7.40, 15.60] | 10.80 [7.50, 15.40] | 10.80 [7.23, 15.88] | 0.868 |  |
| T3(nmol/L) | 1.76 [1.57, 1.98] | 1.75 [1.56, 1.96] | 1.78 [1.59, 2.00] | 0.001 |  |
| TSH(mIU/l) | 2.02 [1.40, 2.97] | 2.02 [1.43, 2.96] | 2.04 [1.36, 2.98] | 0.879 |  |
| FT3(pmol/L) | 4.94 [4.53, 5.34] | 4.93 [4.50, 5.34] | 4.94 [4.56, 5.34] | 0.209 |  |
| FT4(pmol/L) | 16.24 [14.85, 17.80] | 16.30 [14.87, 17.85] | 16.21 [14.83, 17.75] | 0.151 |  |
| T4(nmol/L) | 99.38 [88.26, 111.00] | 98.96 [88.22, 110.20] | 99.70 [88.43, 112.40] | 0.066 |  |
| HCY(umol/L) | 11.42 [8.88, 14.66] | 10.50 [8.06, 13.75] | 12.25 [9.87, 15.59] | <0.001 |  |
| hs-CRP(mg/L) | 0.10 [0.05, 0.19] | 0.08 [0.05, 0.15] | 0.12 [0.07, 0.23] | <0.001 |  |
| sd-LDL-C(mmol/L) | 9.68 [9.44, 9.92] | 9.68 [9.45, 9.88] | 9.69 [9.42, 9.95] | 0.344 |  |
| TyG | 8.72 [8.34, 9.15] | 8.56 [8.17, 8.99] | 8.87 [8.50, 9.28] | <0.001 |  |
| ASBI | 0.06 [0.06, 0.07] | 0.06 [0.06, 0.07] | 0.06 [0.06, 0.07] | 0.176 |  |
| CHG | 5.25 [5.00, 5.50] | 5.15 [4.90, 5.39] | 5.34 [5.10, 5.59] | <0.001 |  |
| NHHR | 2.76 [2.03, 3.58] | 2.56 [1.86, 3.37] | 2.93 [2.20, 3.73] | <0.001 |  |
| RCII | 0.12 [0.04, 0.34] | 0.09 [0.03, 0.24] | 0.17 [0.07, 0.45] | <0.001 |  |
| CALLY | 89.79 [45.30, 155.57] | 107.89 [56.61, 182.66] | 75.49 [37.75, 129.33] | <0.001 |  |
| CLR | 0.05 [0.03, 0.10] | 0.04 [0.02, 0.08] | 0.06 [0.04, 0.12] | <0.001 |  |
| dNLR | 0.88 [0.85, 0.90] | 0.88 [0.86, 0.90] | 0.88 [0.85, 0.90] | 0.048 |  |
| NMLR | 1.98 [1.57, 2.52] | 1.94 [1.55, 2.44] | 2.03 [1.61, 2.60] | <0.001 |  |
| SIRI | 0.56 [0.39, 0.81] | 0.53 [0.37, 0.74] | 0.61 [0.42, 0.85] | <0.001 |  |
| SII | 393.39 [294.96, 524.53] | 392.16 [294.83, 518.42] | 394.08 [295.62, 530.94] | 0.482 |  |
| SHR | 0.84 [0.78, 0.90] | 0.83 [0.79, 0.89] | 0.84 [0.78, 0.91] | 0.125 |  |
| TyG-WHR | 7.87 [7.10, 8.57] | 7.46 [6.66, 8.24] | 8.17 [7.56, 8.80] | <0.001 |  |

Abbreviations: CP, Carotid Plaque; Non-CP, Non-Carotid Plaque; BMI, Body Mass Index; DM, Diabetes Mellitus; MM, Muscle Mass; BMR, Basal Metabolic Rate; WC, Waist Circumference; WHR, Waist-to-Hip Ratio; SBP, Systolic Blood Pressure; DBP, Diastolic Blood Pressure; NLR, Neutrophil-to-Lymphocyte Ratio; MLR, Monocyte-to-Lymphocyte Ratio; PLR, Platelet-to-Lymphocyte Ratio; WBC, White Blood Cell Count; NEUT, Neutrophil Count; LYM, Lymphocyte Count; MONO, Monocyte Count; PLT, Platelet Count; RBC, Red Blood Cell Count; Hb, Hemoglobin; MCV, Mean Corpuscular Volume; MCHC, Mean Corpuscular Hemoglobin Concentration; MCH, Mean Corpuscular Hemoglobin; MPV, Mean Platelet Volume; RDW, Red Cell Distribution Width; Hct, Hematocrit; INR, International Normalized Ratio; PT, Prothrombin Time; PA, Prothrombin Activity; APTT, Activated Partial Thromboplastin Time; FIB, Fibrinogen; PHR, Platelet-to-Hemoglobin Ratio; MHR, Monocyte-to-High-density lipoprotein Cholesterol Ratio; TG, Triglyceride; CHO, Total Cholesterol; HDL-C, High-density Lipoprotein Cholesterol; LDL-C, Low-density Lipoprotein Cholesterol; nonHDL-C, Non-High-Density Lipoprotein Cholesterol; RLP-C, Remnant Lipoprotein Cholesterol; HbA1c, Glycated Hemoglobin; Glu, Glucose; ALT, Alanine Aminotransferase; AST, Aspartate Aminotransferase; ALP, Alkaline Phosphatase; GGT, Gamma-Glutamyl Transferase; TP, Total Protein; ALB, Albumin; TB, Total Bilirubin; DB, Direct Bilirubin; BUN, Blood Urea Nitrogen; Cr, Creatinine; CREA, Creatinine; eGFR, Estimated Glomerular Filtration Rate; UA, Uric Acid; Ca, Calcium; PHOS, Phosphorus; K, Potassium; Na, Sodium; Cl, Chloride; UACR, Urine Albumin-to-Creatinine Ratio; TgAb, Thyroglobulin Antibody; TPOAb, Thyroid Peroxidase Antibody; T3, Triiodothyronine; TSH, Thyroid Stimulating Hormone; FT3, Free Triiodothyronine; FT4, Free Thyroxine; T4, Thyroxine; HCY, Homocysteine; hs-CRP, High-sensitivity C-reactive Protein; sd-LDL-C, Small Dense Low-Density Lipoprotein Cholesterol; TyG, Triglyceride-Glucose Index; ASBI, Atherogenic Index of Plasma; CHG, Cholesterol, high-density lipoprotein, and glucose index; NHHR, Non-HDL-C to HDL-C Ratio; RCII, Residual Cholesterol Ischemic Index; CALLY, C-Reactive Protein-Albumin-Lymphocyte Ratio; CLR, C-Reactive Protein to Lymphocyte Ratio; dNLR, Derived Neutrophil-to-Lymphocyte Ratio; NMLR, Neutrophil-Monocyte-to-Lymphocyte Ratio; SIRI, Systemic Inflammatory Response Index; SII, Systemic Immune-Inflammation Index; SHR, Stress Hyperglycemia Ratio; TyG-WHR, Triglyceride-Glucose-Waist-to-Hip Ratio; .

**Supplementary Table S5. Missing Ratio of Variables in the Study**

| Variable | VIF |
| --- | --- |
| SBP | 1.19725 |
| FIB | 1.081084 |
| MHR | 1.591435 |
| CHO | 1.101754 |
| eGFR | 1.056786 |
| UA | 1.438079 |
| TyG_WHR | 1.989012 |

Abbreviations: VIF, Variance Inflation Factor. SBP, Systolic Blood Pressure; TyG-WHR, Triglyceride-Glucose-Waist-to-Hip Ratio; FIB, Fibrinogen; UA, Uric Acid; eGFR, Estimated Glomerular Filtration Rate; MHR, Monocyte-to-High-density lipoprotein Cholesterol Ratio; CHO, Total Cholestero.

**Supplementary Table S6. Results of nested cross-validation for the full modeling pipeline including recursive feature elimination.**

| Fold | AUC | Best_C | Features |
| --- | --- | --- | --- |
| 1 | 0.818379 | 10 | ['age', 'SBP', 'FIB', 'CHO', 'MHR', 'eGFR', 'UA', 'TyG_WHR'] |
| 2 | 0.772698 | 0.1 | ['age', 'SBP', 'FIB', 'MHR', 'HbA1c', 'eGFR', 'UA', 'TyG_WHR'] |
| 3 | 0.822376 | 1 | ['age', 'SBP', 'FIB', 'CHO', 'MHR', 'eGFR', 'HCY', 'TyG_WHR'] |
| 4 | 0.782253 | 1 | ['age', 'SBP', 'FIB', 'MHR', 'HbA1c', 'eGFR', 'UA', 'TyG_WHR'] |
| 5 | 0.816971 | 0.1 | ['age', 'SBP', 'FIB', 'CHO', 'MHR', 'eGFR', 'HCY', 'TyG_WHR'] |
| 6 | 0.7676 | 1 | ['age', 'SBP', 'FIB', 'MHR', 'GGT', 'eGFR', 'HCY', 'TyG_WHR'] |
| 7 | 0.765977 | 0.1 | ['age', 'SBP', 'RBC', 'FIB', 'MHR', 'eGFR', 'UA', 'TyG_WHR'] |
| 8 | 0.784886 | 0.1 | ['age', 'SBP', 'FIB', 'CHO', 'MHR', 'eGFR', 'UA', 'TyG_WHR'] |
| 9 | 0.772514 | 10 | ['age', 'SBP', 'FIB', 'CHO', 'HbA1c', 'GGT', 'eGFR', 'TyG_WHR'] |
| 10 | 0.80959 | 1 | ['age', 'SBP', 'FIB', 'CHO', 'HbA1c', 'eGFR', 'UA', 'TyG_WHR'] |

Nested cross-validation was performed with 10 outer folds for unbiased estimation of generalization error. Within each outer fold, recursive feature elimination (RFE) with a random forest classifier was applied to the training portion to select the optimal feature subset, followed by hyperparameter tuning of a logistic regression model via grid search with 5-fold inner cross-validation. The average AUC across the 10 outer folds was 0.792, nearly identical to the internal validation set AUC of 0.793 (difference = 0.001), indicating no substantial overfitting due to the feature selection pipeline. AUC, area under the receiver operating characteristic curve; C, inverse regularization strength of the logistic regression model.

| **Supplementary Table S7. Hyperparameter Configurations of the Final Machine Learning Models** | |  |
| --- | --- | --- |
|  |  |  |
| Machine learning models | parameter configuration |  |
| DT | {'ccp_alpha': 0.01, 'max_depth': 3, 'max_features': 'sqrt', 'min_samples_split': 5} |  |
| RF | n_estimators = 50 , max_features = 0.5 |  |
| XGBoost | {'learning_rate': 0.01, 'max_depth': 5, 'n_estimators': 200, 'subsample': 1.0} |  |
| LightGBM | {'colsample_bytree': 0.5, 'learning_rate': 0.05, 'n_estimators': 100, 'num_leaves': 15, 'reg_alpha': 0.1, 'reg_lambda': 0.1, 'subsample': 0.5} |  |
| SVM | {'C': 0.1, 'degree': 2, 'gamma': 'scale', 'kernel': 'rbf'} |  |
| ANN | {'activation': 'relu', 'hidden_layer_sizes': (25,)} |  |

Abbreviations: DT, Decision Tree; RF, Random Forest; XGBoost, Extreme Gradient Boosting; LightGBM, Light Gradient Boosting Machine; SVM, Support Vector Machine; ANN, Artificial Neural Network.

**Supplementary Table S8. P-value Matrix for Pairwise DeLong Test Comparison of Model AUCs**

| **Training set** |  |  |  |  |  |  |  |
| --- | --- | --- | --- | --- | --- | --- | --- |
|  | Logistic | Decision Tree | Random Forest | XGBoost | LightGBM | SVM | ANN |
| Logistic | 1 | 0.0008 | ＜0.001 | ＜0.001 | ＜0.001 | 0.2849 | ＜0.001 |
| Decision Tree | 0.0008 | 1 | ＜0.001 | ＜0.001 | ＜0.001 | 0.0017 | 0.0023 |
| Random Forest | ＜0.001 | ＜0.001 | 1 | ＜0.001 | ＜0.001 | ＜0.001 | ＜0.001 |
| XGBoost | ＜0.001 | ＜0.001 | ＜0.001 | 1 | ＜0.001 | ＜0.001 | ＜0.001 |
| LightGBM | ＜0.001 | ＜0.001 | ＜0.001 | ＜0.001 | 1 | ＜0.001 | ＜0.001 |
| SVM | 0.2849 | 0.0017 | ＜0.001 | ＜0.001 | ＜0.001 | 1 | ＜0.001 |
| ANN | ＜0.001 | 0.0023 | ＜0.001 | ＜0.001 | ＜0.001 | ＜0.001 | 1 |
| **Validation set** |  |  |  |  |  |  |  |
|  | Logistic | Decision Tree | Random Forest | XGBoost | LightGBM | SVM | ANN |
| Logistic | 1 | ＜0.001 | 0.9665 | 0.621 | 0.5071 | 0.8933 | ＜0.001 |
| Decision Tree | ＜0.001 | 1 | ＜0.001 | ＜0.001 | ＜0.001 | ＜0.001 | 0.1289 |
| Random Forest | 0.9665 | ＜0.001 | 1 | 0.6442 | 0.4146 | 0.9433 | ＜0.001 |
| XGBoost | 0.621 | ＜0.001 | 0.6442 | 1 | 0.6533 | 0.6231 | ＜0.001 |
| LightGBM | 0.5071 | ＜0.001 | 0.4146 | 0.6533 | 1 | 0.5158 | ＜0.001 |
| SVM | 0.8933 | ＜0.001 | 0.9433 | 0.6231 | 0.5158 | 1 | ＜0.001 |
| ANN | ＜0.001 | 0.1289 | ＜0.001 | ＜0.001 | ＜0.001 | ＜0.001 | ＜0.001 |
| **Temporal validation set** |  |  |  |  |  |  |  |
|  | Logistic | Decision Tree | Random Forest | XGBoost | LightGBM | SVM | ANN |
| Logistic | 1 | ＜0.001 | 0.8353 | 0.0354 | ＜0.001 | 0.1034 | ＜0.001 |
| Decision Tree | ＜0.001 | 1 | ＜0.001 | ＜0.001 | ＜0.001 | ＜0.001 | 0.8936 |
| Random Forest | 0.8353 | ＜0.001 | 1 | 0.079 | ＜0.001 | 0.6259 | ＜0.001 |
| XGBoost | 0.0354 | ＜0.001 | 0.079 | 1 | ＜0.001 | 0.1379 | ＜0.001 |
| LightGBM | ＜0.001 | ＜0.001 | ＜0.001 | ＜0.001 | 1 | ＜0.001 | ＜0.001 |
| SVM | 0.1034 | ＜0.001 | 0.6259 | 0.1379 | ＜0.001 | 1 | ＜0.001 |
| ANN | ＜0.001 | 0.8936 | ＜0.001 | ＜0.001 | ＜0.001 | ＜0.001 | 1 |

Note: Values in the table represent two-sided P-values from the DeLong test.

**Supplementary Table S9. Conversion of raw variable values to nomogram points**

| Variable | Beta | Range-Min | Range-Max | b_0_ | x1 | Formula |
| --- | --- | --- | --- | --- | --- | --- |
| age | 0.137 | 45.000 | 56.000 | -409.091 | 9.091 | Points = -409.091 + 9.091 × Value |
| SBP | 0.014 | 113.000 | 134.000 | -104.484 | 0.925 | Points = -104.484 + 0.925 × Value |
| TyG-WHR | 0.263 | 7.280 | 8.720 | -126.952 | 17.431 | Points = -126.952 + 17.431 × Value |
| FIB | 0.236 | 2.730 | 3.410 | -42.696 | 15.640 | Points = -42.696 + 15.640 × Value |
| UA | 0.002 | 282.030 | 403.700 | -28.688 | 0.102 | Points = -28.688 + 0.102 × Value |
| eGFR | -0.004 | 93.130 | 107.750 | 30.633 | -0.284 | Points = 30.633 - 0.284 × Value |
| MHR | 0.531 | 0.190 | 0.340 | -6.644 | 35.226 | Points = -6.644 + 35.226 × Value |
| CHO | 0.085 | 2.500 | 3.530 | -14.092 | 5.637 | Points = -14.092 + 5.637 × Value |

Note: SBP, systolic blood pressure; TyG-WHR, triglyceride-glucose-waist-to-hip ratio; FIB, fibrinogen; UA, uric acid; eGFR, estimated glomerular filtration rate; MHR, monocyte-to-HDL cholesterol ratio; CHO, total cholesterol.

**Supplementary Figure**

**Supplementary Figure S1.Assessment of Model Performance on the Training Set**


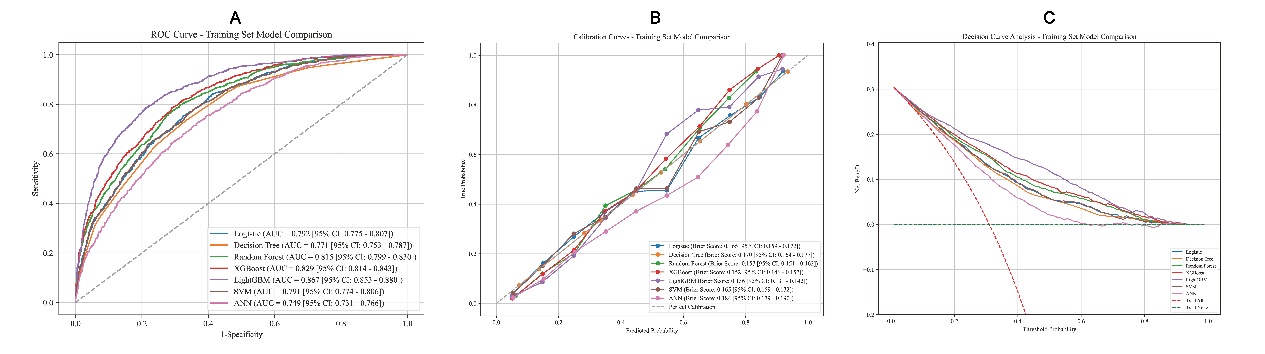


(A) Receiver Operating Characteristic (ROC) curves on the Training Cohort. (B) Calibration curves on the Training Cohort. (C)Decision Curve Analysis (DCA) on the Training Cohort.

Abbreviations: AUC, area under the curve; CI, confidence interval; XGBoost,eXtreme Gradient Boosting; LightGBM,Light gradient boosting machine; SVM, support vector machine; ANN, artificial neural network.
